# Habitat Type Affects Elevational Patterns in Ground-dwelling Arthropod Communities

**DOI:** 10.1093/jisesa/ieac046

**Published:** 2022-08-19

**Authors:** Derek A Uhey, Matthew A Bowker, Karen A Haubensak, David Auty, Sneha Vissa, Richard W Hofstetter

**Affiliations:** School of Forestry, Northern Arizona University, 200 East Pine Knoll Drive, Flagstaff, AZ 86011, USA; School of Forestry, Northern Arizona University, 200 East Pine Knoll Drive, Flagstaff, AZ 86011, USA; Department of Biological Sciences and Center for Ecosystem Science and Society, Northern Arizona University, 617 North Beaver Road, Flagstaff, AZ 86011, USA; School of Forestry, Northern Arizona University, 200 East Pine Knoll Drive, Flagstaff, AZ 86011, USA; School of Forestry, Northern Arizona University, 200 East Pine Knoll Drive, Flagstaff, AZ 86011, USA; School of Forestry, Northern Arizona University, 200 East Pine Knoll Drive, Flagstaff, AZ 86011, USA

**Keywords:** beetle, arachnids, altitude, climate, Colorado Plateau

## Abstract

Understanding factors that drive biodiversity distributions is central in ecology and critical to conservation. Elevational gradients are useful for studying the effects of climate on biodiversity but it can be difficult to disentangle climate effects from resource differences among habitat types. Here we compare elevational patterns and influences of environmental variables on ground-dwelling arthropods in open- and forested-habitats. We examine these comparisons in three arthropod functional groups (detritivores, predators, and herbivores) and two taxonomic groups (beetles and arachnids). We sampled twelve sites spanning 1,132 m elevation and four life zones, collecting 4,834 individual ground arthropods identified to 123 taxa. Elevation was a strong predicator for arthropod composition, however, patterns differed among functional and taxonomic groups and individual species between open- and forested-habitats. Beetles, arachnids, and predators decreased with elevation in open habitats but increased in forests showing a significant interaction between habitat type and elevation. Detritivores and herbivores showed no elevational patterns. We found 11 arthropod taxa with linear elevational patterns, seven that peaked in abundance at high elevations, and four taxa at low elevations. We also found eight taxa with parabolic elevational patterns that peaked in abundance at mid-elevations. We found that vegetation composition and productivity had stronger explanatory power for arthropod composition in forested habitats, while ground cover was a stronger predictor in open habitats. Temperature and precipitation were important in both habitats. Our findings demonstrate that relationships between animal diversity and elevation can be mediated by habitat type, suggesting that physiological restraints and resource limitations work differently between habitat types.

Understanding factors shaping biodiversity distributions has been a long standing goal for ecologists and is critical to conservation efforts (Fattorini et al. 2019). Numerous mechanisms shape biodiversity patterns (see Rahbek 1995), but generally include parts of two hypotheses: physiological tolerance (i.e., direct effects of climate) and resource availability (i.e., indirect effect of climate through productivity) (Sundqvist et al. 2013). The former hypothesizes higher diversity under warmer and wetter conditions, while the latter hypothesizes higher diversity with increased food availability. In wet tropical regions, these hypotheses align at low elevations where temperature and precipitation are highest. However, in arid regions precipitation increases with elevation causing lower elevations to be water-stressed and higher elevations to be temperature-stressed (Rahbek 1995, McCain and Grytnes 2010). Elevational patterns of biodiversity in arid regions can therefore be informative of relationships to climate as species and communities balance precipitation and temperature needs. For arid elevationbal gradients, peaks in diversity are at mid-elevations where preferred climates are located (e.g., Supriya et al. 2019).

Vegetation distribution along elevational gradients is largely shaped by climate (Rahbek 1995), which causes landscape-level patterns in plant species assemblages which partition into distinct communities or ‘life-zones’ consistent with changes in elevation (Merriam 1898). Differences in these plant communities are dramatic when considering the elevational placement of forested and non-forested (open) habitat types. However, this same variation in habitat type can also occur within a life-zone, as forested and open habitats can occur within a single life-zone or range of elevation. Habitat type modulates climate effects on biodiversity, as open habitats are exposed while forested habitats offer some degree of insulation to temperature and precipitation changes (Renaud et al. 2011, Fox et al. 2015). This is demonstrated by habitat types changing elevational patterns of biodiversity. Lasmar et al. (2020) and Uhey et al. (2020a) show that habitat type changed the observed elevational patterns of ant communities. Similar findings are reported for geometrid moths (Axmancher et al. 2009), dung beetles (Mantoni et al. 2021), and skipper moths (Carneiro et al. 2014).

Our study compares patterns of ground-dwelling arthropods (i.e., beetles, arachnids, true-bugs, and others) in two contrasting habitat types (forested and open) across two elevational gradients on the southern Colorado Plateau. Arid biogeographical regions like the Colorado Plateau, along with ground arthropod groups, are underrepresented in the elevational literature, but are integral to ecosystem structure. Previous research on ants in this system found biodiversity increased with elevation in open habitats but decreased with elevation in forested habitats (Uhey et al. 2020a). Compared to ants which are highly thermophilic and are mostly omnivorous (Weiser and Kaspari 2006), other arthropod groups (e.g., beetles) may be more cold tolerant (Sinclair 1999) and/or more reliant on specific vegetation compositions (e.g., herbivores, Whitham et al. 2006). This may cause the effects of habitat type on elevational patterns to change depending on which arthropod groups are considered. Here we examine the effect of habitat type on elevational patterns of ground-dwelling arthropod functional groups (e.g., herbivores, predators, and detritivores) and taxonomic groups (e.g., beetles and arachnids). We ask: 1) do elevational patterns of arthropods change between habitat types? 2) which environmental variables (climate or vegetation) are the most influential on arthropod diversity in forested versus open habitats? We hypothesized that habitat type would be more important for groups specialized to vegetation composition (e.g., herbivores) compared to generalist groups (e.g., detritivores). We also hypothesized that climate would have a more direct impact in open habitats, while forested habitats would be affected more strongly by vegetation.

## Materials and Methods

### Study Design

**Fig. 1.**
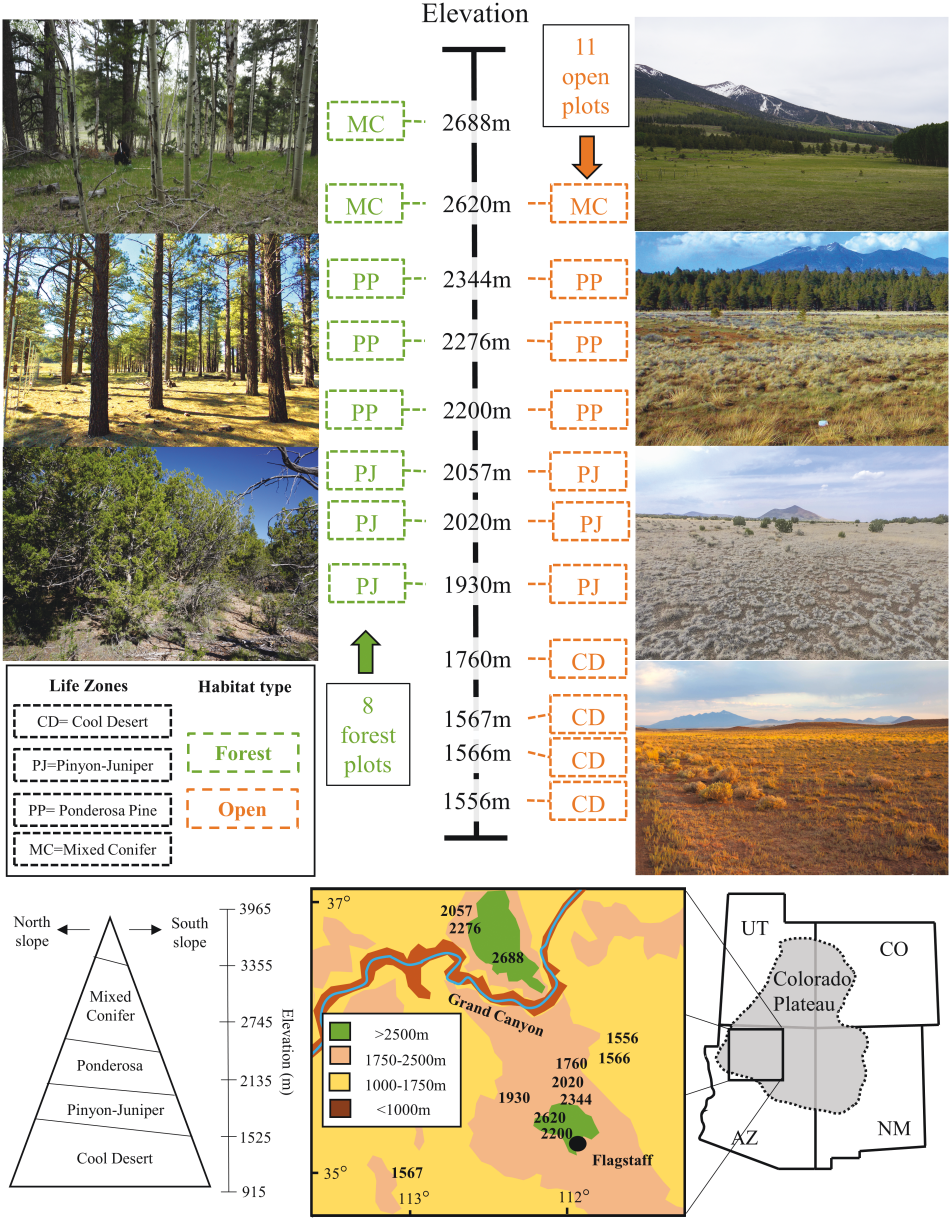
Locations (stated as elevation in meters, e.g., 2,276 m), photos, and sampling scheme of elevational gradient sites in northern Arizona on the southwestern Colorado Plateau. Twelve sites spanned four life zones (Cool Desert, Pinyon-Juniper, Ponderosa, and Mixed Conifer), with forested and open plots paired where possible, totaling nine forested and eleven open plots.

We conducted our study in northern Arizona using 12 sites (Fig. 1, Supp Table 1 [online only]) from two established elevational gradients (C. Hart Merriam Elevational Gradient (MEG) [Wu et al. 2011]) and the Southwestern Experimental Garden Array (SEGA, sega.nau.edu). For the purpose of this study we merged all sites into a single elevational gradient. Collectively, these sites span 1,556–2,688 m in elevation, encompassing four life zones: cool desert (CD), pinyon-juniper (PJ), ponderosa pine (PP), and mixed conifer (MC). Climate and vegetation of these sites are detailed at sega.nau.edu. In short, CD sites are semi-arid grasslands or shrublands with no trees; PJ sites are woodlands dominated by pinyon [*Pinus edulis* (Engelm., Pinaceae: Pinales)] and juniper [*Juniperus* spp. (L., Cupressaceae: Pinales)] trees with interspersed open areas dominated by grasses and shrubs; PP sites are forests dominated by ponderosa pine [*Pinus ponderosa* (Douglas, Pinaceae: Pinales)] with open meadow areas dominated by grasses; and MC sites are forests dominated by aspen [*Populus tremuliodes* (Michx., Salicaceae: Malpighiales)], white fir [*Abies concolor* (Lindley, Pinaceae: Pinales)], Douglas fir [*Pseudotsuga menziesii* (Franco, Pinaceae: Pinales)], and blue spruce [*Picea pungens* (Engelm., Pinaceae: Pinales)] with small meadows dominated by grasses and forbs. Along our gradient, elevation correlated strongly with average annual precipitation (*r* = 0.90, *p* < 0.001) and temperature (*r* = −0.96, *p* < 0.001), with average annual precipitation increasing from 127 mm/year at the lowest site to 772 mm/year at the highest site, and average annual temperature decreasing from 13.6 to 6.7°C (Supp Table 1 [online only]).

We established 30 m by 30m plots at each site, pairing plots (>200 m to <1 km apart) at sites that offered both open- and forest-habitats (total of seven of the twelve sites). The highest site had only forest habitat available and all CD sites had only open habitats available, thus single plots were established at these five sites (Fig. 1, Supp Table 1 [online only]). We purposefully placed plots in relatively undisturbed areas that best represented either open or forest habitats. At each of the 19 plots (seven pairs, five singles), we sampled arthropods and vegetation within five replicate 1m^2^ subplots. We positioned subplots with one in the center and the other four located halfway between the center and the corners of the large plot such that all subplots were separated at least 10 m from one another and from the edge of the 900 m^2^ plot (Supp Fig. 1 [online only]). We used on-site weather stations supplemented with field temperature loggers to collect temperature and precipitation data from each plot during each sampling period.

### Arthropod Sampling

To sample arthropods, we used pitfall traps with a single trap dug at the center of each subplot left open for two 7-day sampling periods during the dry (9–16 June 2015) and monsoon (6–13 August 2015) seasons. These two summer seasons encompass the active periods for most arthropods in our region. We constructed traps following Higgins et al. (2014). In short, each trap consisted of a borosilicate glass tube (32 mm in diameter and 200 mm in length) filled with ~100 ml of propylene glycol and fitted into a PVC sleeve with a rain cover to allow arthropods to enter the trap. We chose these traps due to key advantages over larger diameter traps:1) lower evaporation rates so they can be left out longer (our sampling period was 1-week),2) less likely to capture or have preservative consumed by nontarget vertebrate species,3) more likely to sample litter dwelling species that do not surface often (traps have lids that sit low allowing litter crawlers to enter without exposure to sunlight), and4) PVC-tubing holds pit-trap placement constant between sampling periods (Higgins et al. 2014). Our traps commonly sampled insects as large as 25 mm [e.g., *Eleodes obscurus* (Say, Tenebrionidae: Coleoptera)]. We collected from 89/95 traps during the dry season and 75/95 during the monsoon season as some traps were lost to flooding or wildlife damage. We sorted and identified specimens with specialist help, and deposited voucher specimens at the Colorado Museum of Arthropod Biodiversity at Northern Arizona University, and cataloged specimen images on Symbiota Collections of Arthropod Network (https://scan-bugs.org/portal/) and bugguide.net (Supp Table 1 [online only]). Patterns of ants (Formicidae) in this system are reported by Uhey et al. (2020a); here we focus on patterns of other ground-dwelling arthropod groups.

We assigned taxonomic designations to individuals with the assistance of experts to the lowest practical taxonomic level (e.g., Cecil et al. 2019, Ferrenberg et al. 2019, Hasin and Booncher 2020). Ecological patterns are often similar when compared among different taxonomic resolutions, becoming more apparent at species-level (see Timms et al. 2013 and Gerwing et al. 2020). Therefore our approach is conservative and may underestimate differences. We identified 57.8% of specimens to species/morphospecies-level, 16.5% to genus-level, 18.6% to family-level, and 7.2% to order-level. We identified some groups (e.g., Coleoptera, Hymenoptera, Orthoptera, and Hemiptera) to a higher taxonomic resolution than others (e.g., arachnids) because of differences in taxonomic resources (i.e., experts). Hereafter we refer to our mixed-bag of lowest possible taxonomic-levels as ‘taxa richness’ (equivalent to ‘lowest practical taxonomic units’, e.g., Coccia and Farina 2019, De Jong et al. 2021). We excluded from our analyses but report in Supplemental Material: Diptera, Lepidoptera, and parasitic Apocrita (Hymenoptera) as nontarget taxa; larval Coleoptera, and nymphs of Hemiptera and Araneae which could not be identified; and springtails in the family Entomobryidae which were extremely variable in abundance. We assigned each taxon to one of three functional groups (predators, herbivores, and detritivores) determined by their trophic interactions.

### Environmental Variables

To understand the relationship of plant composition and cover to arthropods, we measured vegetation during both dry and monsoon sampling periods by using point-intercept of 25 points within a gridded 1 m^2^ quadrat for each subplot (Godinez-Alvarez et al. 2009). We categorized points as vegetation species/morphospecies with 63 in total including 33 forbs, 19 grasses, 8 shrubs, and 3 tree species (Supp Table 1 [online only]). We estimated site-level productivity with normalized difference vegetation index (NDVI) calculated from satellite imagery (Pettorelli et al. 2005). NDVI ranges from zero to one with higher values indicating higher productivity (Pettorelli et al. 2011). We downloaded data at a spatial resolution of 800 m for each sampled date range and extracted data using R version 3.2.3 and the package ‘raster’ version 2.5-2 based on observed latitude and longitude of each site and the date range of our arthropod sampling. While the resolution of 800 m is larger than our plot size, it is still informative of climate among sites which are widely spaced (>5 km).

We compiled environmental measurements into the following predictor variables: 30-year average annual temperature, 30-year average annual precipitation, NDVI, mean temperature during sampling, precipitation during sampling, vegetation richness, ground cover percent (also broken into grass and forb percents), and vegetation composition. Vegetation composition was modeled with Bray–Curtis similarity coefficients calculated from vegetation data, with two sets of non-metric multidimensional scaling (NMDS) ordination coordinates representing composition (stress = 0.12), hereafter referred to as ‘Veg1’ and ‘Veg2’. Veg1 was driven by variation in several grasses [*Elymus elymoides* (Swezey, Poaceae: Poales)*, Festuca arizonica* (Hackel, Poaceae: Poales), *Sporobolus airoides* (Torr., Poaceae: Poales)] and the small shrub *Guiterezzia microphylla* (Gray, Asteraceae: Asterales). Veg2 was driven by variation in non-native cheatgrass [*Bromus tectorum* (L., Poaceae: Poales)], *Bouteloua gracilis* (Lag., Poaceae: Poales), *Mahonia aquifolium* (Nutt., Berberidaceae: Ranunculales), and *Pinus ponderosae*.

## Analysis

To evaluate inventory completeness of each site, we calculated sample coverage. For each site we characterized Hill numbers (i.e., Hill order (q)) to quantify biodiversity: 1) total taxonomic richness (i.e., q = 0), 2) exponential of Shannon’s entropy index (i.e., *q* = 1), and 3) inverse Simpson’s concentration index (i.e., *q* = 2). We calculated these for incidence based data and extrapolated or interpolated values to double the minimum sample size (*n* = 10) and created 95% confidence intervals using asymptotic Chao1 estimators (Chao et al. 2014). We calculated sample coverage using the function *iNext* and extrapolations/interpolations of diversity using the function *estimateD* in the R package ‘iNext’ (Hsieh et al. 2016). To account for differences in sample sizes, we conducted all our subsequent analyses at the level of individual pitfall trap measurements. We conducted all analyses in R.3.6.2. and provide model details and R code in Supplemental File 1.

### Habitat Effects on Elevational Patterns

To test if habitat type alters elevational patterns of ground-dwelling arthropod diversity, we used generalized linear mixed-effects models (GLMM). We made separate models with the taxa richness and abundance of five arthropod groups (detritivores, predators, herbivores, beetles, and arachnids) as response variables. We included site as a random effect in each model to account for spatial dependencies of pitfall traps. For each response variable, we tested Poisson and negative binomial distributions, with or without zero-inflation. We used likelihood ratio tests and Akaike information criterion (AIC) to choose the best distribution. Taxa richness always followed Poisson distributions without zero inflation, while abundance always followed negative binomial distributions with some cases of zero inflation (Supplemental File 1).

We compared models with all combinations of elevation, habitat, and date as fixed effects (Table 1), using likelihood tests and AIC to select the best performing model for each arthropod response variable. Elevational patterns can be linear (y = a + bx), parabolic (y = a + bx^2^), or quadratic (y = a + bx + cx^2^) (Werenkraut and Ruggiero 2014), therefore we compared linear, squared, and quadratic elevation terms to find the best possible elevational pattern to explain arthropod data. To address our hypothesis that habitats affect elevational patterns of arthropods, we modeled habitat as an interaction with elevation terms. We report final models and significant predictors (alpha = 0.05) with model details available in Supplemental File 1. We used the function *glmmTMB* to construct and compare all GLMMs and the function *predict* to make 95% confidence intervals of model predictions, both from the R package ‘glmmTMB’ (Brooks et al. 2017).

**Table 1.** GLMMs compared for best fit to data using combinations of predictor variables: elevation (linear, parabolic, and quadratic terms), habitat type (open vs forest), and season (dry vs monsoon) with site as a random effect for each of ten arthropod response variables (detritivore richness, detritivore abundance, predator richness, predator abundance, herbivore richness, herbivore abundance, beetle richness, beetle abundance, arachnid richness, and arachnid abundance)

Model type	Model formula
Null	y ~ 1 + (1|Site)
Habitat only	y ~ Habitat + (1|Site)
Season only	y ~ Season + (1|Site)
Habitat plus season	y ~ Habitat + Season + (1|Site)
Elevation (linear)	y ~ Elevation + (1|Site)
Elevation (linear) plus season	y ~ Elevation + Season + (1|Site)
Elevation (linear) interaction with habitat	y ~ Elevation * Habitat + (1|Site)
Elevation (linear) interaction with habitat plus season	y ~ Elevation * Habitat + Season + (1|Site)
Elevation (parabolic)	y ~ Elevation^2^ + (1|Site)
Elevation (parabolic) plus season	y ~ Elevation^2^ + Season + (1|Site)
Elevation (parabolic) interaction with habitat	y ~ Elevation^2^ * Habitat + (1|Site)
Elevation (parabolic) interaction with habitat plus season	y ~ Elevation^2^ * Habitat + Season + (1|Site)
Elevation (quadratic)	y ~ Elevation + Elevation^2 + (1|Site)
Elevation (quadratic) plus season	y ~ Elevation + Elevation^2 + Season + (1|Site)
Elevation (quadratic) interaction with habitat	y ~ Elevation + Elevation^2 * Habitat + (1|Site)
Elevation (quadratic) interaction with habitat plus season	y ~ Elevation + Elevation^2 * Habitat + Season + (1|Site)

We selected the best performing model using AIC for each of ten arthropod response variables (detritivore richness, detritivore abundance, predator richness, predator abundance, herbivore richness, herbivore abundance, beetle richness, beetle abundance, arachnid richness, and arachnid abundance).

To test if habitat type alters elevational patterns of individual ground-dwelling arthropod taxa, we used multivariate generalized linear models (GLMs) with function *manyglm* in R-package ‘mvabund’ (Wang et al. 2012). *Manyglms* fits a single GLM to abundance data for each species, giving individual taxa estimates of significance while controlling for multiple testing. We compared the same models as the above GLMMs (Table 1), using AIC to select the best elevational model to describe our data. We report significant predictor variables (alpha = 0.05) for individual taxa from the best model (see Supp Table 2 [online only] for model details).

### Environmental Variable Relationships in Open and Forest Habitats

We created NMDS ordinations to visualize patterns in our data and facilitate further analysis. To test effects of environmental variables in habitat types, we compared correlations of environmental variables to NMDS ordination axes based on Bray–Curtis similarity coefficients calculated from arthropod data in either open or forested habitats. We used goodness of fit, Shepard diagrams, and ordination stress to confirm satisfactory fit of ordination. Elevational patterns in beta-diversity are often categorized by life zone. For each habitat type, we tested if arthropods grouped by life zone by using permutational analysis of variance (PERMANOVA, permutations = 9999). We fitted environmental variables to ordinations via correlations to any possible axis and used permutations to test significance with the R packages ‘vegan’ (Oksanen et al. 2013) and ‘ecodist’ (Goslee and Urban 2007). For all analyses, code and output can be found in Supplemental File 1.

## Results

In total, we collected 4,834 individual ground-dwelling arthropods from 123 taxa. Functionally, 2,357 individuals in 46 taxa were detritivores, 1,447 individuals in 51 taxa were predators, and 1,030 individuals in 27 taxa were herbivores. Taxonomically, 1,743 individuals in 61 taxa were Coleoptera, 1,150 individuals in 25 taxa were Arachnida, 833 individuals in 13 taxa were Hemiptera, and 282 individuals in 7 taxa were Orthoptera. The remaining 826 individuals were in 17 taxa and are hereafter referred to as ‘others’.

**Fig. 2.**
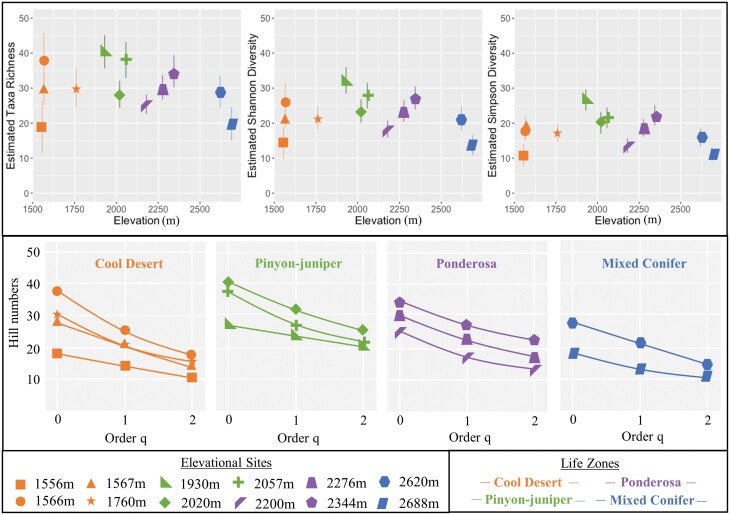
Top panel: Estimated Hill numbers (i.e., taxa richness [*q* = 0], exponential Shannon index [*q* = 1], and inverse Simpson index [*q* = 2] diversities) for twelve elevational sites (shapes) in four life zones with 95% confidence intervals (lines). Bottom panel: Diversity curves of Hill numbers (i.e., order *q*) across elevational sites (shapes) in four life zones.

Sample coverage was high, ranging among sites from 74.5 to 95.6% (see Supplemental File 1 for statistic details, and Supp Fig. 2 [online only] for rarifacation curves). In general, diversity peaked at middle elevation sites (Fig. 2). The highest diversity was seen in two sites in the pinyon-juniper life zone (1,930 m and 2,057 m) and one site in the cool desert life zone (1,566 m) while the lowest diversity was found at both highest (2,688 m) and lowest (1,556 m) elevations. A drop in Hill orders moving from *q* = 0 to *q* = 2 indicates unevenness (i.e., dominance of single species, Chao et al. 2014). The larger this drop the larger the unevenness. Our data showed similar trends among sites of moderate decreases across Hill orders, indicating some unevenness at all elevations (Fig. 2).

### Elevational Patterns of Arthropod Diversity in Different Habitat Types

Elevational patterns were significant in taxa richness and abundance in three arthropod groups: beetles, arachnids, and predators (Table 2). Season was also a significant predictor for these groups, with increases in taxa richness and abundance during the monsoon. Beetle and arachnid richness followed linear elevational patterns, while beetle and arachnid abundance followed quadratic patterns. Predators also followed quadratic elevational patterns. Herbivores and detritivores had no significant relationships with elevation or any predictors, except for herbivore richness which was significantly higher in open habitat.

**Table 2.** The best models with significant (*p* < 0.05) predictors for 10 arthropod response variables (model specifics available in Supplemental File 1)

Response	Best model	Significant predictors (*p* < 0.05)
Beetle richness	y ~ Elevation*Habitat + Season	Elevation (linear), Habitat, Elevation*Habitat, Season
Beetle abundance	y ~ Elevation + Elevation^2*Habitat + Season	Elevation (quadratic), Elevation*Habitat, Season
Arachnid richness	y ~ Elevation	Elevation (linear)
Arachnid abundance	y ~ Elevation + Elevation^2*Habitat + Season	Elevation (quadratic), Elevation*Habitat, Season
Predator richness	y ~ Elevation + Elevation^2*Habitat	Elevation (quadratic), Elevation*Habitat, Season
Predator abundance	y ~ Elevation + Elevation^2*Habitat	Elevation (quadratic), Elevation*Habitat, Season
Herbivore richness	y ~ Habitat	Habitat
Herbivore abundance	Null model	None
Detritivore richness	Null model	None
Detritivore abundance	Null model	None

All models included site as a random effect. Model specifics are available in Supplemental File 1.

**Fig. 3.**
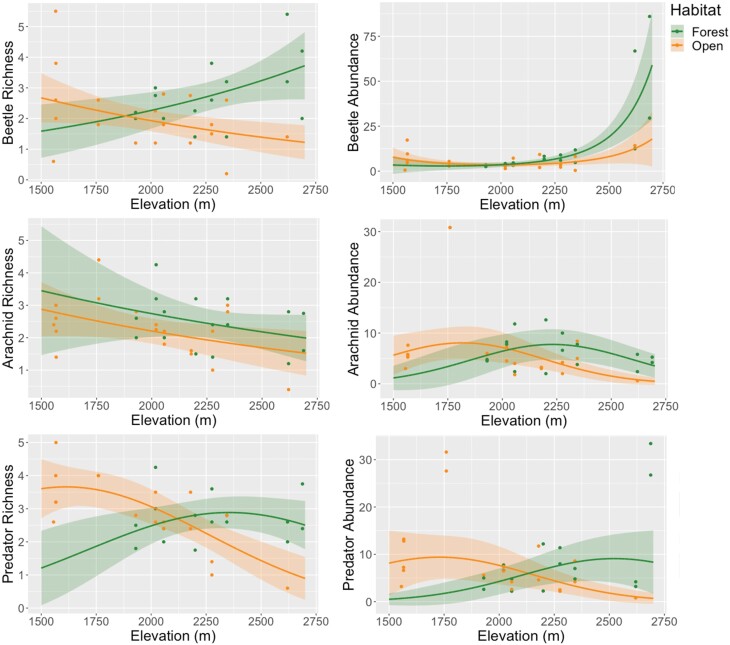
Three arthropod groups (beetles, arachnids, and predators) measured as taxa richness (left-hand panels) and abundance (right-hand panels) with significant elevational patterns in two habitat types: open and forested. Circles show actual averages of data, while lines show predicted values from GLMMs with shaded areas showing 95% confidence intervals for predictions.

The taxa richness and abundance of beetles and predators, as well as arachnid abundance, had significant interactions of habitat type and elevation (Table 2). In general, these measures increased with elevation in forested habitats, but decreased with elevation in open habitats (Fig. 3). In these cases, the highest taxa richness or abundance was found at high elevations in forests, and at low elevations in open habitats. Arachnid richness decreased with elevation similarly between habitat types.

Individual taxa responded differently to elevation with 11 showing linear elevation patterns and eight showing parabolic elevational patterns (Table 3). Seven taxa with linear elevation patterns peaked in abundance at high elevations (i.e., mixed conifer), while the other four peaked at low elevations (i.e., cool desert). The eight taxa with parabolic elevational patterns peaked in abundance at mid-elevations (i.e., either pinyon-juniper or ponderosa life zones). Habitat was a significant predictor for seven taxa with five taxa more abundant in forests and two taxa more abundant in open habitats. Two taxa showed significant interactions of habitat on elevational patterns: Orbatid mites which were most abundant at low elevations in open habitats, and Julida millipedes which were most abundant at high elevation in open habitats. Season was a significant predictor for five taxa (Table 3).

**Table 3.** Ground-dwelling arthropods as a function of season, elevation, and/or habitat type (CD = cool desert, PJ = pinyon-juniper, PP = ponderosa pine, MC = mixed conifer, O = open, F = forest) as determined by GLM analysis (only significant relationships (*p* < 0.05) shown, all taxa results in Supplemental Table 2)

Taxa (lowest identification level)	Taxonomic group	Functional group	Significant predictors (*p* < 0.05)	Life zone, habitat peak
Anystidae	Arachnid	Predator	Elevation (linear)	CD-O
Microtrombidiidae	Arachnid	Predator	Elevation (linear)	MC-F
Lycosidae	Arachnid	Predator	Elevation (parabolic)	PP-F
Eremobatidae	Arachnid	Predator	Elevation (parabolic), Season	CD-O
Orbatida	Arachnid	Predator	Elevation*Habitat, Season	CD-O
Bdellidae	Arachnid	Predator	Habitat	PJ-F
*Chlaenius tomentosus*	Beetle	Predator	Elevation (linear)	CD-O
*Pasimachus californicus*	Beetle	Predator	Elevation (linear)	CD-O
*Eleodes rileyi*	Beetle	Detritivore	Elevation (linear)	MC-F
*Pterostichus protractus*	Beetle	Predator	Elevation (linear), Habitat	MC-F
Staphylinidae: Aleocharinae	Beetle	Detritivore	Elevation (linear), Habitat	MC-F
*Synuchus dubius*	Beetle	Predator	Elevation (linear), Season	MC-F
Ptiliidae	Beetle	Detritivore	Elevation (linear), Season	MC-F
*Thalycra*sp.	Beetle	Detritivore	Elevation (parabolic)	PP-F
*Eleodes obscurus*	Beetle	Detritivore	Elevation (parabolic)	PJ-O
*Notoxus nuperus*	Beetle	Predator	Elevation (parabolic), Season	PJ-F
*Epuraea* sp.	Beetle	Detritivore	Habitat	PP-F
*Eleodes hispilabris*	Beetle	Detritivore	Habitat	CD-O
*Coelocnemis magna*	Beetle	Detritivore	Habitat	PJ-F
Microcoryphia	Bristletail	Detritivore	Elevation (parabolic)	PJ-O
*Emblethis vicarius*	Hemipteran	Detritivore	Elevation (linear)	CD-O
*Stachyocnemus apicalis*	Hemipteran	Herbivore	Elevation (parabolic)	PJ-O
Aphididae	Hemipteran	Herbivore	Elevation (parabolic)	PP-F
Julida	Millipede	Detritivore	Elevation (linear), Elevation*Habitat	MC-O
*Melanoplus*sp.	Orthopteran	Herbivore	Habitat	PJ-O

Only taxa with significant relationships (*p* < 0.05) to elevation (linear term), elevation^2^ (parabolic term), both elevation terms (quadratic), season, or habitat type are shown. The life zone and habitat combination with the highest abundance are given for each taxa (CD = cool desert, PJ = pinyon-juniper, PP = ponderosa pine, MC = mixed conifer, O = open, F = forest).

### Environmental Effects in Forested Versus Open Habitats

**Fig. 4.**
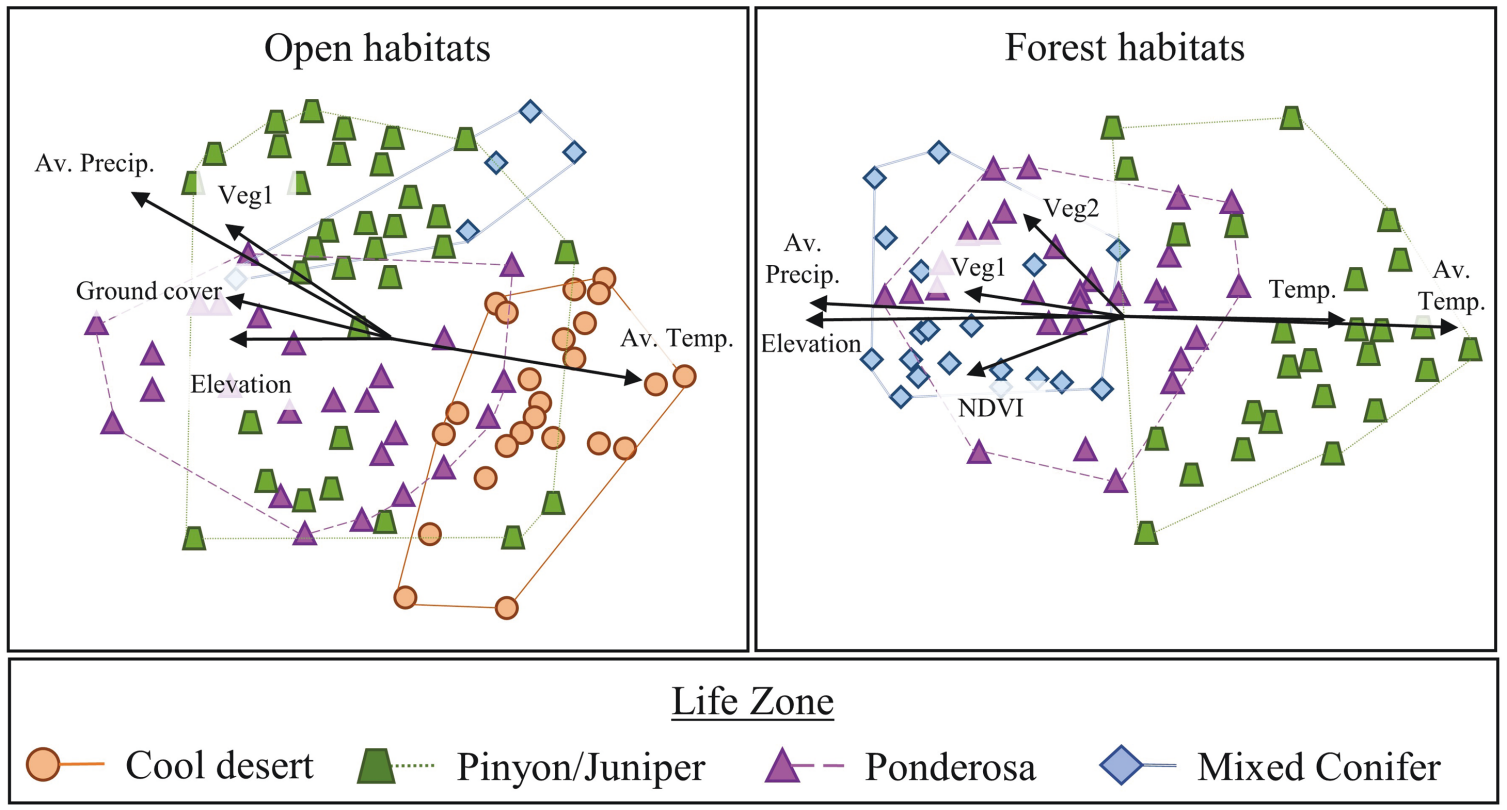
Ordination of ground-dwelling arthropod communities of open- and forested-sites in four life zones (shape). Each point represents arthropod composition from a pitfall trap. Arrows show significant (*p* < 0.05) correlation of environmental variables (Supplemental Table 2).

Arthropod communities significantly grouped by life zone in both forested (F_2,71_ = 5.86, *p* < 0.001) and open (F_3,85_ = 3.80, *p* < 0.001) habitats (Fig. 4). Arthropod composition (i.e., NMDS axes) in forested habitats showed stronger correlations with elevation (*R*^2^_open_ = 0.10, *p*_open_ = 0.015; *R*^2^_forest_ = 0.58, *p*_forest_ < 0.001), average annual temperature (*R*^2^_open_ = 0.24, *p*_open_ < 0.001; *R*^2^_forest_ = 0.57, *p*_forest_ < 0.001), and precipitation (*R*^2^_open_ = 0.32, *p*_open_ < 0.001; *R*^2^_forest_ = 0.57, *p*_forest_ < 0.001) than open habitats. Arthropod composition significantly correlated with NDVI (i.e., productivity, *R*^2^_forest_ = 0.19, *p*_forest_ = 0.002), temperature measured during sampling, and Veg2 (axis two of vegetation NMDS, *R*^2^_forest_ = 0.19, *p*_forest_ < 0.001) only in forested habitats. Arthropod composition significantly correlated with ground cover percent (*R*^2^_open_ = 0.09, *p*_open_ = 0.025) only in open habitats. Arthropod composition in both habitats had moderate correlations with Veg1 (axis one of vegetation NMDS, *R*^2^_open_ = 0.13, *p*_open_ = 0.006; *R*^2^_forest_ = 0.15, *p*_forest_ = 0.005). Precipitation during sampling, vegetation richness, and grass or forb cover did not correlate with arthropod composition (Supp Table 2 [online only]).

## Discussion

We found that ground-dwelling arthropods present different elevational patterns in open- versus forested-habitat types. Beetles, arachnids, and predators increased with elevation in forested habitats but decreased in open habitats, showing similar patterns to ants (Uhey et al. 2020a). The modulation of elevational responses by habitat type suggests arthropods are differentially affected by climate factors and/or resource availability in forested versus open habitats.

Habitat type and climate are often correlated along elevational gradients, as the former can be driven by the latter (Rahbek 1995). This study allowed us to partially disentangle these effects by pairing forested and open sites. Despite the limitation of our study in underrepresenting some species and therefore conservatively estimating patterns, we found many interesting relationships of elevation and habitat with arthropod groups and species. In many cases modeling of arthropod patterns was improved by incorporating habitat type interactions with elevation. This demonstrates how habitat type is important to consider when interpretating elevational patterns of arthropods and inferring climate mechanisms. Also important to consider are the various forms of elevational patterns (linear, parabolic, and quadratic) as we found cases of each.

### Arthropod Elevational Patterns and Habitat Type

Why do arthropods in forests tend to have positive relationships with elevation while having negative relationships in open habitats? The answer may lay in differences in available resources (Fox et al. 2015) and mediation of climate by habitat (Renaud et al. 2011). Forests are more productive, retain higher moisture, and better insulate temperature changes than open habitats (Renaud et al. 2011). Forests also generally offer more refugia and resources than open habitats which are exposed to climate extremes and are more physiologically stressful for organisms (Sthultz et al. 2007, Michalet et al. 2014, Cuautle et al. 2016). Areas of greatest climate stress for arthropods may therefore occur at different elevational ranges along open- versus forested-gradients. For example, arthropods may struggle with cold or drought in open habitats but find refuge from these in forested habitats.

Arthropods likely need to balance temperature and precipitation needs differentially between habitat types affecting which environmental variables are most important in forests or open habitats. We found vegetation composition and productivity were more associated with arthropods in forested rather than open habitats. This indicates that plant-arthropod interactions may be stronger in shaping communities in forested habitats, which are generally more productive than open habitats. Oppositely, ground cover percent was associated with arthropods in open, but not forested habitats. The effect of ground cover may be less in forested habitats as trees shade out plants, while open habitats usually have an abundance of ground cover.

### Climate and Arthropods

Regardless of habitat type, elevation, temperature, and precipitation were strongly correlated with arthropod composition showing the strong role of climate in our system. The links between arthropods and climate are well-established (e.g., Sanders et al. 2007; Donoso et al. 2010; Meyer et al. 2015, Uhey et al. 2020a, b, 2021; Vissa et al. 2021). Many ground-dwelling arthropods are detritivores with general diets, likely not specialized to specific plant species (Illig et al. 2005, Donoso et al. 2010, Uhey et al. 2020c). However, these arthropods are desiccation-prone and thermophilic causing physiological dependence on specific climates (Hodkinson 2005). This is reflected in the strong predictive power of temperature for arthropod composition in our study.

In arid systems such as the Colorado Plateau, where this study was conducted, precipitation is also a particularly strong driver acting as a limiting trophic currency (Allen et al. 2014). We accordingly found precipitation to be a strong predictor of arthropod composition, similar to other arid elevation gradients (e.g., Cuautle et al. 2016, Gonzalez-Reyes et al. 2017, Supriya et al. 2019). Temperature and precipitation are the dominant forces shaping communities into life zones (Merriam 1898). We therefore found ground-dwelling arthropod communities grouped well under the life zone concept, similar to other biota in our region such as bees (McCabe et al. 2019, 2020, 2021; Chesshire et al. 2021), birds (Bock and Webb 1984), ants (Uhey et al. 2020a), and plants (Merriam 1898, Fernandes 1992).

Arthropod groups responded differently to elevation which can give insights into their relationships with climate. Many groups and individual arthropod taxa peaked at mid-elevations, indicating preferred climates where arthropods can balance temperature and precipitation needs. Arachnids generally decreased with elevation, an indication that temperature is a stronger driver for this largely predacious group. This is supported by predators typically being more successful in warmer environments (Broitman et al. 2009). Oppositely, beetles generally increased with elevation; an indication that precipitation (which is higher at higher elevations in arid regions) is a strong driver for this group. Consequently, beetles made up over 90% of specimens in the high-elevation mixed conifer life zone. This was largely driven by increases in predacious ground beetles (Carabidae) and detritivorous rove beetles (Staphylinidae). Both these families of beetles are common in colder more humid climates at high elevations (Pakeman et al. 2014), potentially finding competitive release from ants that dominate warmer climates at low elevations (Olson 1994, Uhey et al. 2021).

Across the southwestern United States, average annual temperatures are predicted to increase 1.5–2°C by the year 2050 causing prolonged droughts (Karmalkar and Bradley 2017). Already dramatic shifts in the vegetation structure are occurring largely driven by tree mortality in low-elevation forests, which will potentially shift the elevational ranges of life zones or create novel ecosystems (Cayan et al. 2010, Minott and Kolb 2020). Our results suggest these trends are likely to extend to ground-dwelling arthropods, either directly through physiological responses or indirectly through changes to vegetation structure. Shifts in arthropod communities driven by changes in vegetation structure from climate change may subsequently result in cascading trophic effects on species richness on macroecological scales (Ferger et al. 2014).

## Conclusion

We found that habitat type can modulate elevational patterns of arthropod diversity, suggesting differences in resource limitations or physiological restraints between open- and forested-habitats. Forest cover may partially override or modify underlying physiological mechanisms inducing stronger vegetation control of arthropod communities, while open habitats may have stronger relationships to climate. Habitat-type may be particularly influential on how arthropods balance precipitation and temperature needs. The positive relationship of arthropod diversity with elevation in forests suggests these habitats are precipitation limited, while converse elevational patterns in open-habitats suggest temperature limitations. Some arthropod groups may be more sensitive to habitat type than others because of their specific resource or climate requirements, and we recommend future studies test open- and forested-elevational patterns in additional taxa. We conclude that fundamental aspects of climate-animal relationships are mediated by habitat structure which is therefore an important factors in shaping biodiversity patterns.

## Supplementary Material

ieac046_suppl_Supplementary_Fig_1

ieac046_suppl_Supplementary_Fig_2

ieac046_suppl_Supplementary_File_1

ieac046_suppl_Supplementary_Table_1

ieac046_suppl_Supplementary_Table_2
