# Supplementary figures and images for: Habitat Type Affects Elevational Patterns in Ground-dwelling Arthropod Communities
Source: J Insect Sci. 2022 Aug 19;22(4):9. doi: 10.1093/jisesa/ieac046 (PMC11639851; doi:10.1093/jisesa/ieac046)

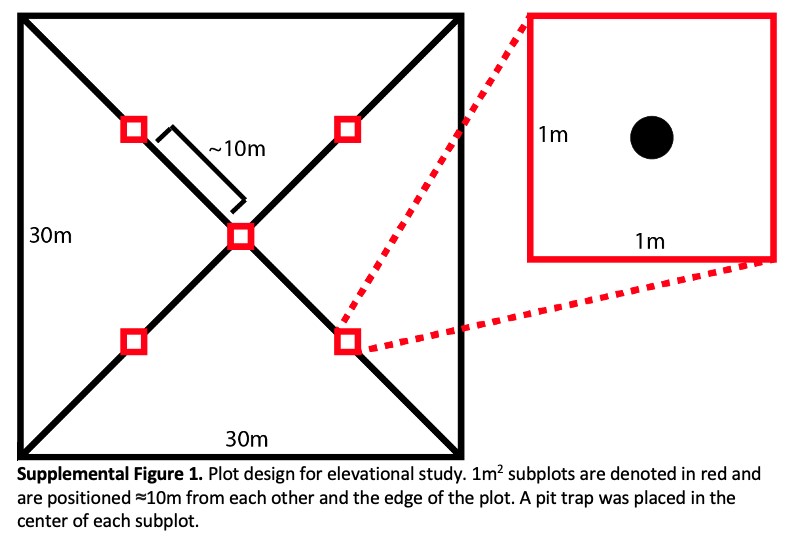

Supplement: ieac046_suppl_Supplementary_Fig_1 [file ieac046_suppl_supplementary_fig_1.jpeg]

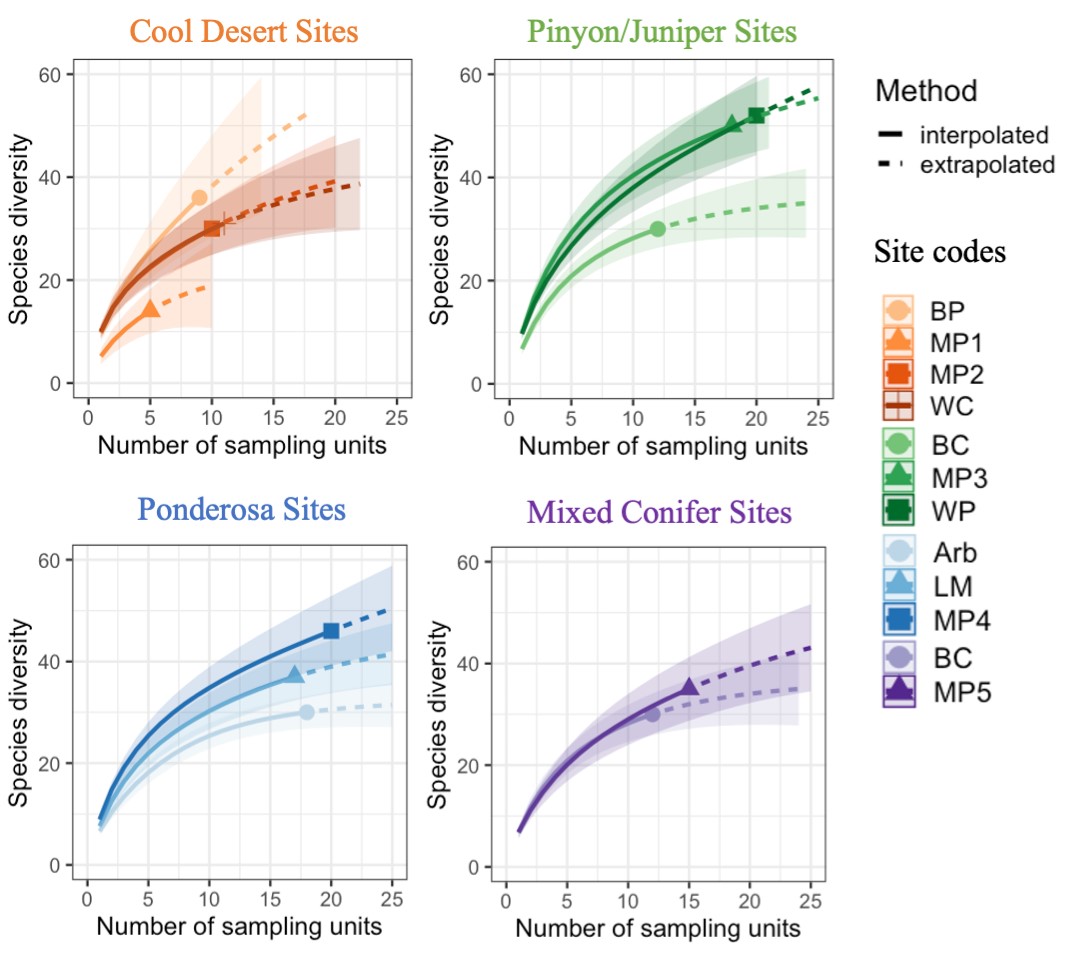

Supplement: ieac046_suppl_Supplementary_Fig_2 [file ieac046_suppl_supplementary_fig_2.jpeg]
