# Supplementary material for: Habitat Type Affects Elevational Patterns in Ground-dwelling Arthropod Communities
Source: J Insect Sci. 2022 Aug 19;22(4):9. doi: 10.1093/jisesa/ieac046 (PMC11639851; doi:10.1093/jisesa/ieac046)
Supplement: ieac046_suppl_Supplementary_File_1 [file ieac046_suppl_supplementary_file_1.html]

Habitat type affects elevational patterns in ground-dwelling arthropod communities


# Habitat type affects elevational patterns in ground-dwelling arthropod communities

#### Derek Uhey

```
#load libraries
library(iNEXT)
library(ggplot2)
library(devtools)
```

```
## Loading required package: usethis
```

```
## Error in get(genname, envir = envir) : object 'testthat_print' not found
```

```
library(gridExtra)
library("writexl")
library(tidyverse)
```

```
## ── Attaching packages ───────────────────────────────────────────────────────────────────────────────────────────────── tidyverse 1.3.0 ──
```

```
## ✓ tibble  3.0.3     ✓ dplyr   1.0.0
## ✓ tidyr   1.1.0     ✓ stringr 1.4.0
## ✓ readr   1.4.0     ✓ forcats 0.5.0
## ✓ purrr   0.3.4
```

```
## ── Conflicts ──────────────────────────────────────────────────────────────────────────────────────────────────── tidyverse_conflicts() ──
## x dplyr::combine() masks gridExtra::combine()
## x dplyr::filter()  masks stats::filter()
## x dplyr::lag()     masks stats::lag()
```

```
library(glmmTMB)
```

```
## Warning: package 'glmmTMB' was built under R version 4.0.5
```

```
## Warning in checkMatrixPackageVersion(): Package version inconsistency detected.
## TMB was built with Matrix version 1.4.1
## Current Matrix version is 1.2.18
## Please re-install 'TMB' from source using install.packages('TMB', type = 'source') or ask CRAN for a binary version of 'TMB' matching CRAN's 'Matrix' package
```

```
## Warning in checkDepPackageVersion(dep_pkg = "TMB"): Package version inconsistency detected.
## glmmTMB was built with TMB version 1.8.0
## Current TMB version is 1.8.1
## Please re-install glmmTMB from source or restore original 'TMB' package (see '?reinstalling' for more information)
```

```
library(performance)
```

```
## Warning: package 'performance' was built under R version 4.0.5
```

```
library(MuMIn)
```

```
## Warning: package 'MuMIn' was built under R version 4.0.5
```

```
library(mvabund)
```

```
## 
## Attaching package: 'mvabund'
```

```
## The following object is masked from 'package:MuMIn':
## 
##     coefplot
```

```
library(vegan)
```

```
## Loading required package: permute
```

```
## 
## Attaching package: 'permute'
```

```
## The following object is masked from 'package:devtools':
## 
##     check
```

```
## Loading required package: lattice
```

```
## This is vegan 2.5-6
```

```
#Datasheets
sega <- read.csv("Library/Mobile Documents/com~apple~CloudDocs/NAU/Masters Work/2015/2015.csv", header = TRUE)
sega_f <-read.csv("Library/Mobile Documents/com~apple~CloudDocs/NAU/Masters Work/2015/2015_Forest.csv", header = TRUE)
sega_o <-read.csv("Library/Mobile Documents/com~apple~CloudDocs/NAU/Masters Work/2015/2015_Open.csv", header = TRUE)
#iNext datasheets for each arthropod group
Det <-read.csv("Library/Mobile Documents/com~apple~CloudDocs/NAU/Masters Work/2015/JIS_submission/Det_iNext.csv", header = TRUE)
Det <-Det[2:20]
Pred <-read.csv("Library/Mobile Documents/com~apple~CloudDocs/NAU/Masters Work/2015/JIS_submission/Pred_iNext.csv", header = TRUE)
Pred <-Pred[2:20]
Beet <-read.csv("Library/Mobile Documents/com~apple~CloudDocs/NAU/Masters Work/2015/JIS_submission/Beet_iNext.csv", header = TRUE)
Beet <-Beet[2:20]
ACH <-read.csv("Library/Mobile Documents/com~apple~CloudDocs/NAU/Masters Work/2015/JIS_submission/Arach_iNext.csv", header = TRUE)
ACH <-ACH[2:20]
#Datasheets of arthropods by site for iNext analysis
Art <-read.csv("Library/Mobile Documents/com~apple~CloudDocs/NAU/Masters Work/2015/JIS_submission/Arthropods.csv", header = TRUE)
Art <-Art[2:13]
R <-read.csv("Library/Mobile Documents/com~apple~CloudDocs/NAU/Masters Work/2015/JIS_submission/Rich.csv", header = TRUE)
SH<-read.csv("Library/Mobile Documents/com~apple~CloudDocs/NAU/Masters Work/2015/JIS_submission/Shannon.csv", header = TRUE)
SP<-read.csv("Library/Mobile Documents/com~apple~CloudDocs/NAU/Masters Work/2015/JIS_submission/Simpson.csv", header = TRUE)
```

```
#iNext analysis for sample coverage and hill numbers
DataInfo(Art, datatype="incidence_freq")
```

```
estimateD(Art, datatype="incidence_freq", base="size", level=20)
```

```
#Accumulation curves
#Cool Desert life zone curves
CD <- select(Art, MP1, MP2, BP, WC)
out <- iNEXT(CD, datatype="incidence_freq", endpoint=NULL)
ggiNEXT(out, type=1, color.var="site") + 
  theme_bw(base_size = 18) + 
  theme(legend.position="right") +
  xlim(0,25) + ylim(0,60) +
  scale_color_manual(values=c("#fdbe85", "#fd8d3c", "#e6550d", "#a63603")) + scale_fill_manual(values=c("#fdbe85", "#fd8d3c", "#e6550d", "#a63603"))
```

```
#Pinyon Juniper life zone curves
PJ <- select(Art, BC, MP3, WP)
out <- iNEXT(PJ, datatype="incidence_freq", endpoint=NULL)
ggiNEXT(out, type=1, color.var="site") + 
  theme_bw(base_size = 18) + 
  theme(legend.position="right") +
  xlim(0,25) + ylim(0,60) +
  scale_color_manual(values=c("#74c476", "#31a354", "#006d2c")) + scale_fill_manual(values=c("#74c476", "#31a354", "#006d2c"))
```

```
## Warning: Removed 27 row(s) containing missing values (geom_path).
```

```
#Ponderosa life zone curves
PP <- select(Art, Arb, LM, MP4)
out <- iNEXT(PP, datatype="incidence_freq", endpoint=NULL)
ggiNEXT(out, type=1, color.var="site") + 
  theme_bw(base_size = 18) + 
  theme(legend.position="right") +
  xlim(0,25) + ylim(0,60) +
  scale_color_manual(values=c("#bdd7e7", "#6baed6", "#2171b5")) + scale_fill_manual(values=c("#bdd7e7", "#6baed6", "#2171b5"))
```

```
## Warning: Removed 37 row(s) containing missing values (geom_path).
```

```
#Mixed Conifer life zone curves
MC <- select(Art, MP5, BC)
out <- iNEXT(MC, datatype="incidence_freq", endpoint=NULL)
ggiNEXT(out, type=1, color.var="site") + 
  theme_bw(base_size = 18) + 
  theme(legend.position="right") +
  xlim(0,25) + ylim(0,60) +
  scale_color_manual(values=c("#9e9ac8", "#54278f")) + scale_fill_manual(values=c("#9e9ac8", "#54278f"))
```

```
## Warning: Removed 6 row(s) containing missing values (geom_path).
```

```
#GLMM variables
#Make scaled elevation term for modeling
sega$elescale <- (sega$Elevation - mean(sega$Elevation))/sd(sega$Elevation)
elescale <- (sega$Elevation - mean(sega$Elevation))/sd(sega$Elevation)
# Change Date to a factor
unique(sega$Date)
```

```
## [1] "June 9-16, 2015"   "August 6-13, 2015"
```

```
sega$Date <- as.factor(sega$Date)
levels(sega$Date)
```

```
## [1] "August 6-13, 2015" "June 9-16, 2015"
```

```
# Make Date into new Season variable:
sega <- sega %>%
  mutate(Season = recode(Date,
                         "June 9-16, 2015" = "Dry",
                         "August 6-13, 2015" = "Monsoon"),
         Season = fct_relevel(Season, c("Dry","Monsoon")))
levels(sega$Season)
```

```
## [1] "Dry"     "Monsoon"
```

```
#Make prediction data for graphing
newdat <- expand.grid(Elevation = seq(1500, 2700, 10), Habitat = unique(sega$Habitat), Site = unique(sega$Site), Season = unique(sega$Season))
newdat$elescale = (newdat$Elevation - 2112.141)/329.1319
```

```
#Beetle_Richness
#Family test, poisson versus neg.binomial and zero-inflated 
Pmodel = glmmTMB(Beetle_Richness ~ 1  + (1 | Site),
                 ziformula = ~0,
                 family = poisson(link = "log"),
                 data = sega)
Pmodel_z = glmmTMB(Beetle_Richness ~ 1  + (1 | Site),
                   ziformula = ~1,
                   family = poisson(link = "log"),
                   data = sega)
Nmodel = glmmTMB(Beetle_Richness ~ 1  + (1 | Site),
                 ziformula = ~0,
                 family = nbinom2(link = "log"),
                 data = sega)
```

```
## Warning in fitTMB(TMBStruc): Model convergence problem; false convergence (8).
## See vignette('troubleshooting')
```

```
Nmodel_z = glmmTMB(Beetle_Richness ~ 1  + (1 | Site),
                   ziformula = ~1,
                   family = nbinom2(link = "log"),
                   data = sega)
```

```
## Warning in fitTMB(TMBStruc): Model convergence problem; false convergence (8).
## See vignette('troubleshooting')
```

```
anova(Pmodel, Pmodel_z, Nmodel, Nmodel_z)
```

```
#Find Best Model
N = glmmTMB(Beetle_Richness ~ 1  + (1 | Site),
            ziformula = ~0,
            family = poisson(link = "log"),
            data = sega)
H = glmmTMB(Beetle_Richness ~ Habitat + (1 | Site),
            ziformula = ~0,
            family = poisson(link = "log"),
            data = sega)
E = glmmTMB(Beetle_Richness ~ elescale  + (1 | Site),
            ziformula = ~0,
            family = poisson(link = "log"),
            data = sega)
E2 = glmmTMB(Beetle_Richness ~ I(elescale^2) + (1 | Site),
             ziformula = ~0,
             family = poisson(link = "log"),
             data = sega)
EE2 = glmmTMB(Beetle_Richness ~ elescale + I(elescale^2) + (1 | Site),
              ziformula = ~0,
              family = poisson(link = "log"),
              data = sega)
EH = glmmTMB(Beetle_Richness ~ elescale * Habitat  + (1 | Site),
             ziformula = ~0,
             family = poisson(link = "log"),
             data = sega)
E2H = glmmTMB(Beetle_Richness ~ I(elescale^2) * Habitat  + (1 | Site),
              ziformula = ~0,
              family = poisson(link = "log"),
              data = sega)
EE2H = glmmTMB(Beetle_Richness ~ elescale * Habitat + I(elescale^2) + (1 | Site),
               ziformula = ~0,
               family = poisson(link = "log"),
               data = sega)
HD = glmmTMB(Beetle_Richness ~ Habitat + Season + (1 | Site),
             ziformula = ~0,
             family = poisson(link = "log"),
             data = sega)
ED = glmmTMB(Beetle_Richness ~ elescale  + Season + (1 | Site),
             ziformula = ~0,
             family = poisson(link = "log"),
             data = sega)
E2D = glmmTMB(Beetle_Richness ~ I(elescale^2) + Season + (1 | Site),
              ziformula = ~0,
              family = poisson(link = "log"),
              data = sega)
EE2D = glmmTMB(Beetle_Richness ~ elescale + I(elescale^2) + Season + (1 | Site),
               ziformula = ~0,
               family = poisson(link = "log"),
               data = sega)
EHD = glmmTMB(Beetle_Richness ~ elescale * Habitat  + Season + (1 | Site),
              ziformula = ~0,
              family = poisson(link = "log"),
              data = sega)
E2HD = glmmTMB(Beetle_Richness ~ I(elescale^2) * Habitat  + Season + (1 | Site),
               ziformula = ~0,
               family = poisson(link = "log"),
               data = sega)
EE2HD = glmmTMB(Beetle_Richness ~ elescale * Habitat + I(elescale^2) + Season + (1 | Site),
                ziformula = ~0,
                family = poisson(link = "log"),
                data = sega)
D = glmmTMB(Beetle_Richness ~ Date + (1 | Site),
            ziformula = ~0,
            family = poisson(link = "log"),
            data = sega)
anova(N, H, E, E2, EE2, EH, E2H, EE2H, HD, ED, E2D, EE2D, EHD, E2HD, EE2HD, D)
```

```
#Best model statistics and predictions
summary(EHD)
```

```
##  Family: poisson  ( log )
## Formula:          Beetle_Richness ~ elescale * Habitat + Season + (1 | Site)
## Data: sega
## 
##      AIC      BIC   logLik deviance df.resid 
##    580.6    599.1   -284.3    568.6      157 
## 
## Random effects:
## 
## Conditional model:
##  Groups Name        Variance Std.Dev.
##  Site   (Intercept) 0.008037 0.08965 
## Number of obs: 163, groups:  Site, 20
## 
## Conditional model:
##                      Estimate Std. Error z value Pr(>|z|)    
## (Intercept)            0.7915     0.1112   7.117  1.1e-12 ***
## elescale               0.2203     0.0966   2.281 0.022559 *  
## HabitatOpen           -0.3079     0.1353  -2.275 0.022897 *  
## SeasonMonsoon          0.2215     0.1061   2.087 0.036862 *  
## elescale:HabitatOpen  -0.4605     0.1273  -3.617 0.000298 ***
## ---
## Signif. codes:  0 '***' 0.001 '**' 0.01 '*' 0.05 '.' 0.1 ' ' 1
```

```
r.squaredGLMM(EHD)
```

```
## Warning: 'r.squaredGLMM' now calculates a revised statistic. See the help page.
```

```
## Warning in r.squaredGLMM.glmmTMB(EHD): the effects of zero-inflation and
## dispersion model are ignored
```

```
## Warning: the null model is correct only if all variables used by the original
## model remain unchanged.
```

```
##                 R2m       R2c
## delta     0.1556541 0.1716013
## lognormal 0.1801819 0.1986421
## trigamma  0.1301187 0.1434498
```

```
#Get average values of actual data for graph
sega2 <- sega %>%
  group_by(Season, Site, Habitat, Elevation) %>%
  summarise(Averages = mean(Beetle_Richness))
```

```
## `summarise()` regrouping output by 'Season', 'Site', 'Habitat' (override with `.groups` argument)
```

```
# Make new dataframe for prediction:
newdat$beetle_pred <- predict(EH, newdata = newdat, type = "response", re.form=NULL)
preds <- predict(EH, newdat, se=T, allow.new.levels = T, type='response', re.form=~0)
newdat$pred = preds$fit
newdat$se = preds$se.fit
newdat$ulimit = newdat$pred + 1.96*newdat$se
newdat$llimit = newdat$pred - 1.96*newdat$se
#Plot Results, observed data as points, predicted values from model as trend lines
ggplot(data=newdat, aes(x = Elevation, y = pred))+
  geom_ribbon(aes(ymin=llimit, ymax=ulimit, fill = Habitat), alpha = 0.25)  +
  geom_point(aes(x = Elevation, y = Averages, color = Habitat), data=sega2) +
  geom_line(aes(color = Habitat), size=1) +
  labs(x = "Elevation (m)", y = "Beetle Richness") + 
  scale_color_manual(values=c("#228B22", "#FF8C00")) + scale_fill_manual(values=c("#228B22", "#FF8C00")) +
  theme(text = element_text(size = 20))
```

```
#Beetle_Abundance
#Family test, poisson versus neg.binomial and zero-inflated 
Pmodel = glmmTMB(Beetle_Abundance ~ 1  + (1 | Site),
                 ziformula = ~0,
                 family = poisson(link = "log"),
                 data = sega)
Pmodel_z = glmmTMB(Beetle_Abundance ~ 1  + (1 | Site),
                   ziformula = ~1,
                   family = poisson(link = "log"),
                   data = sega)
Nmodel = glmmTMB(Beetle_Abundance ~ 1  + (1 | Site),
                 ziformula = ~0,
                 family = nbinom2(link = "log"),
                 data = sega)
Nmodel_z = glmmTMB(Beetle_Abundance ~ 1  + (1 | Site),
                   ziformula = ~1,
                   family = nbinom2(link = "log"),
                   data = sega)
anova(Pmodel, Pmodel_z, Nmodel, Nmodel_z)
```

```
#Find Best Model
N = glmmTMB(Beetle_Abundance ~ 1  + (1 | Site),
            ziformula = ~1,
            family = nbinom2(link = "log"),
            data = sega)
D = glmmTMB(Beetle_Abundance ~ Season  + (1 | Site),
            ziformula = ~1,
            family = nbinom2(link = "log"),
            data = sega)
H = glmmTMB(Beetle_Abundance ~ Habitat + (1 | Site),
            ziformula = ~1,
            family = nbinom2(link = "log"),
            data = sega)
E = glmmTMB(Beetle_Abundance ~ elescale  + (1 | Site),
            ziformula = ~1,
            family = nbinom2(link = "log"),
            data = sega)
E2 = glmmTMB(Beetle_Abundance ~ I(elescale^2) + (1 | Site),
             ziformula = ~1,
             family = nbinom2(link = "log"),
             data = sega)
EE2 = glmmTMB(Beetle_Abundance ~ elescale + I(elescale^2) + (1 | Site),
              ziformula = ~1,
              family = nbinom2(link = "log"),
              data = sega)
EH = glmmTMB(Beetle_Abundance ~ elescale * Habitat  + (1 | Site),
             ziformula = ~1,
             family = nbinom2(link = "log"),
             data = sega)
E2H = glmmTMB(Beetle_Abundance ~ I(elescale^2) * Habitat  + (1 | Site),
              ziformula = ~1,
              family = nbinom2(link = "log"),
              data = sega)
EE2H = glmmTMB(Beetle_Abundance ~ elescale * Habitat + I(elescale^2) + (1 | Site),
               ziformula = ~1,
               family = nbinom2(link = "log"),
               data = sega)
HD = glmmTMB(Beetle_Abundance ~ Habitat + Season + (1 | Site),
             ziformula = ~1,
             family = nbinom2(link = "log"),
             data = sega)
ED = glmmTMB(Beetle_Abundance ~ elescale  + Season + (1 | Site),
             ziformula = ~1,
             family = nbinom2(link = "log"),
             data = sega)
E2D = glmmTMB(Beetle_Abundance ~ I(elescale^2) + Season + (1 | Site),
              ziformula = ~1,
              family = nbinom2(link = "log"),
              data = sega)
EE2D = glmmTMB(Beetle_Abundance ~ elescale + I(elescale^2) + Season + (1 | Site),
               ziformula = ~1,
               family = nbinom2(link = "log"),
               data = sega)
EHD = glmmTMB(Beetle_Abundance ~ elescale * Habitat  + Season + (1 | Site),
              ziformula = ~1,
              family = nbinom2(link = "log"),
              data = sega)
E2HD = glmmTMB(Beetle_Abundance ~ I(elescale^2) * Habitat  + Season + (1 | Site),
               ziformula = ~1,
               family = nbinom2(link = "log"),
               data = sega)
EE2HD = glmmTMB(Beetle_Abundance ~ elescale * Habitat + I(elescale^2) + Season + (1 | Site),
                ziformula = ~1,
                family = nbinom2(link = "log"),
                data = sega)
anova(N, D, H, E, E2, EE2, EH, E2H, EE2H, HD, ED, E2D, EE2D, EHD, E2HD, EE2HD)
```

```
#Best model statistics and predictions
summary(EE2HD)
```

```
##  Family: nbinom2  ( log )
## Formula:          
## Beetle_Abundance ~ elescale * Habitat + I(elescale^2) + Season +  
##     (1 | Site)
## Zero inflation:                    ~1
## Data: sega
## 
##      AIC      BIC   logLik deviance df.resid 
##    972.3   1000.1   -477.1    954.3      154 
## 
## Random effects:
## 
## Conditional model:
##  Groups Name        Variance  Std.Dev. 
##  Site   (Intercept) 9.067e-09 9.522e-05
## Number of obs: 163, groups:  Site, 20
## 
## Dispersion parameter for nbinom2 family (): 2.28 
## 
## Conditional model:
##                      Estimate Std. Error z value Pr(>|z|)    
## (Intercept)           1.36312    0.13116  10.392  < 2e-16 ***
## elescale              0.80982    0.16277   4.975 6.52e-07 ***
## HabitatOpen          -0.12340    0.17424  -0.708 0.478807    
## I(elescale^2)         0.32202    0.08406   3.831 0.000128 ***
## SeasonMonsoon         0.40959    0.13543   3.024 0.002491 ** 
## elescale:HabitatOpen -0.62099    0.22173  -2.801 0.005101 ** 
## ---
## Signif. codes:  0 '***' 0.001 '**' 0.01 '*' 0.05 '.' 0.1 ' ' 1
## 
## Zero-inflation model:
##             Estimate Std. Error z value Pr(>|z|)    
## (Intercept)  -2.7527     0.5528   -4.98 6.37e-07 ***
## ---
## Signif. codes:  0 '***' 0.001 '**' 0.01 '*' 0.05 '.' 0.1 ' ' 1
```

```
r.squaredGLMM(EE2HD)
```

```
## Warning in r.squaredGLMM.glmmTMB(EE2HD): the effects of zero-inflation and
## dispersion model are ignored
```

```
## Warning: the null model is correct only if all variables used by the original
## model remain unchanged.
```

```
##                 R2m       R2c
## delta     0.5318958 0.5318958
## lognormal 0.5863292 0.5863292
## trigamma  0.4642503 0.4642503
```

```
#Get average values of actual data for graph
sega2 <- sega %>%
  group_by(Season, Site, Habitat, Elevation) %>%
  summarise(Averages = mean(Beetle_Abundance))
```

```
## `summarise()` regrouping output by 'Season', 'Site', 'Habitat' (override with `.groups` argument)
```

```
# Make new dataframe for prediction:
newdat$beetle_pred <- predict(EE2H, newdata = newdat, type = "response", re.form=NULL)
preds <- predict(EE2H, newdat, se=T, allow.new.levels = T, type='response', re.form=~0)
newdat$pred = preds$fit
newdat$se = preds$se.fit
newdat$ulimit = newdat$pred + 1.96*newdat$se
newdat$llimit = newdat$pred - 1.96*newdat$se
#Plot Results, observed data as points, predicted values from model as trend lines
ggplot(data=newdat, aes(x = Elevation, y = pred))+
  geom_ribbon(aes(ymin=llimit, ymax=ulimit, fill = Habitat), alpha = 0.25)  +
  geom_point(aes(x = Elevation, y = Averages, color = Habitat), data=sega2) +
  geom_line(aes(color = Habitat), size=1) +
  labs(x = "Elevation (m)", y = "Beetle Abundance") + 
  scale_color_manual(values=c("#228B22", "#FF8C00")) + scale_fill_manual(values=c("#FF8C00", "#228B22")) +
  theme(text = element_text(size = 20))
```

```
#Arachnid_richness
#Family test, poisson versus neg.binomial and zero-inflated 
Pmodel = glmmTMB(Arachnid_richness ~ 1  + (1 | Site),
                 ziformula = ~0,
                 family = poisson(link = "log"),
                 data = sega)
Pmodel_z = glmmTMB(Arachnid_richness ~ 1  + (1 | Site),
                   ziformula = ~1,
                   family = poisson(link = "log"),
                   data = sega)
Nmodel = glmmTMB(Arachnid_richness ~ 1  + (1 | Site),
                 ziformula = ~0,
                 family = nbinom2(link = "log"),
                 data = sega)
```

```
## Warning in fitTMB(TMBStruc): Model convergence problem; false convergence (8).
## See vignette('troubleshooting')
```

```
Nmodel_z = glmmTMB(Arachnid_richness ~ 1  + (1 | Site),
                   ziformula = ~1,
                   family = nbinom2(link = "log"),
                   data = sega)
```

```
## Warning in fitTMB(TMBStruc): Model convergence problem; false convergence (8).
## See vignette('troubleshooting')
```

```
anova(Pmodel, Pmodel_z, Nmodel, Nmodel_z)
```

```
#Find Best Model
N = glmmTMB(Arachnid_richness ~ 1  + (1 | Site),
            ziformula = ~0,
            family = poisson(link = "log"),
            data = sega)
H = glmmTMB(Arachnid_richness ~ Habitat   + (1 | Site),
            ziformula = ~0,
            family = poisson(link = "log"),
            data = sega)
E = glmmTMB(Arachnid_richness ~ elescale + (1 | Site),
            ziformula = ~0,
            family = poisson(link = "log"),
            data = sega)
E2 = glmmTMB(Arachnid_richness ~ I(elescale^2) + (1 | Site),
             ziformula = ~0,
             family = poisson(link = "log"),
             data = sega)
EE2 = glmmTMB(Arachnid_richness ~ elescale + I(elescale^2) + (1 | Site),
              ziformula = ~0,
              family = poisson(link = "log"),
              data = sega)
EH = glmmTMB(Arachnid_richness ~ elescale * Habitat  + (1 | Site),
             ziformula = ~0,
             family = poisson(link = "log"),
             data = sega)
E2H = glmmTMB(Arachnid_richness ~ I(elescale^2) * Habitat  + (1 | Site),
              ziformula = ~0,
              family = poisson(link = "log"),
              data = sega)
EE2H = glmmTMB(Arachnid_richness ~ elescale * Habitat + I(elescale^2) + (1 | Site),
               ziformula = ~0,
               family = poisson(link = "log"),
               data = sega)
HD = glmmTMB(Arachnid_richness ~ Habitat  + Season + (1 | Site),
             ziformula = ~0,
             family = poisson(link = "log"),
             data = sega)
ED = glmmTMB(Arachnid_richness ~ elescale + Season + (1 | Site),
             ziformula = ~0,
             family = poisson(link = "log"),
             data = sega)
E2D = glmmTMB(Arachnid_richness ~ I(elescale^2) + Season + (1 | Site),
              ziformula = ~0,
              family = poisson(link = "log"),
              data = sega)
EE2D = glmmTMB(Arachnid_richness ~ elescale + I(elescale^2) + Season + (1 | Site),
               ziformula = ~0,
               family = poisson(link = "log"),
               data = sega)
EHD = glmmTMB(Arachnid_richness ~ elescale * Habitat  + Season + (1 | Site),
              ziformula = ~0,
              family = poisson(link = "log"),
              data = sega)
E2HD = glmmTMB(Arachnid_richness ~ I(elescale^2) * Habitat  + Season + (1 | Site),
               ziformula = ~0,
               family = poisson(link = "log"),
               data = sega)
EE2HD = glmmTMB(Arachnid_richness ~ elescale * Habitat + I(elescale^2) + Season + (1 | Site),
                ziformula = ~0,
                family = poisson(link = "log"),
                data = sega)
D = glmmTMB(Arachnid_richness ~ Season + (1 | Site),
                ziformula = ~0,
                family = poisson(link = "log"),
                data = sega)
anova(N, D, H, E, E2, EE2, EH, E2H, EE2H, HD, ED, E2D, EE2D, EHD, E2HD, EE2HD)
```

```
#Best model statistics and predictions
summary(E)
```

```
##  Family: poisson  ( log )
## Formula:          Arachnid_richness ~ elescale + (1 | Site)
## Data: sega
## 
##      AIC      BIC   logLik deviance df.resid 
##    563.3    572.6   -278.7    557.3      160 
## 
## Random effects:
## 
## Conditional model:
##  Groups Name        Variance Std.Dev.
##  Site   (Intercept) 0.03082  0.1756  
## Number of obs: 163, groups:  Site, 20
## 
## Conditional model:
##             Estimate Std. Error z value Pr(>|z|)    
## (Intercept)  0.84011    0.06695  12.549   <2e-16 ***
## elescale    -0.11298    0.06515  -1.734   0.0829 .  
## ---
## Signif. codes:  0 '***' 0.001 '**' 0.01 '*' 0.05 '.' 0.1 ' ' 1
```

```
r.squaredGLMM(E)
```

```
## Warning in r.squaredGLMM.glmmTMB(E): the effects of zero-inflation and
## dispersion model are ignored
```

```
## Warning: the null model is correct only if all variables used by the original
## model remain unchanged.
```

```
##                  R2m        R2c
## delta     0.02730218 0.09322392
## lognormal 0.03215156 0.10978222
## trigamma  0.02240614 0.07650628
```

```
#Get average values of actual data for graph
sega2 <- sega %>%
  group_by(Season, Site, Habitat, Elevation) %>%
  summarise(Averages = mean(Arachnid_richness))
```

```
## `summarise()` regrouping output by 'Season', 'Site', 'Habitat' (override with `.groups` argument)
```

```
# Make new dataframe for prediction:
newdat$beetle_pred <- predict(EH, newdata = newdat, type = "response", re.form=NULL)
preds <- predict(EH, newdat, se=T, allow.new.levels = T, type='response', re.form=~0)
newdat$pred = preds$fit
newdat$se = preds$se.fit
newdat$ulimit = newdat$pred + 1.96*newdat$se
newdat$llimit = newdat$pred - 1.96*newdat$se
#Plot Results, observed data as points, predicted values from model as trend lines
ggplot(data=newdat, aes(x = Elevation, y = pred))+
  geom_ribbon(aes(ymin=llimit, ymax=ulimit, fill = Habitat), alpha = 0.25)  +
  geom_point(aes(x = Elevation, y = Averages, color = Habitat), data=sega2) +
  geom_line(aes(color = Habitat), size=1) +
  labs(x = "Elevation (m)", y = "Arachnid Richness") + 
  scale_color_manual(values=c("#228B22", "#FF8C00")) + scale_fill_manual(values=c("#FF8C00", "#228B22")) +
  theme(text = element_text(size = 20))
```

```
#Arachnid_abundance
#Family test, poisson versus neg.binomial and zero-inflated 
Pmodel = glmmTMB(Arachnid_abundance ~ 1  + (1 | Site),
                 ziformula = ~0,
                 family = poisson(link = "log"),
                 data = sega)
Pmodel_z = glmmTMB(Arachnid_abundance ~ 1  + (1 | Site),
                   ziformula = ~1,
                   family = poisson(link = "log"),
                   data = sega)
Nmodel = glmmTMB(Arachnid_abundance ~ 1  + (1 | Site),
                 ziformula = ~0,
                 family = nbinom2(link = "log"),
                 data = sega)
Nmodel_z = glmmTMB(Arachnid_abundance ~ 1  + (1 | Site),
                   ziformula = ~1,
                   family = nbinom2(link = "log"),
                   data = sega)
anova(Pmodel, Pmodel_z, Nmodel, Nmodel_z)
```

```
#Find Best Model
N = glmmTMB(Arachnid_abundance ~ 1  + (1 | Site),
            ziformula = ~0,
            family = nbinom2(link = "log"),
            data = sega)
H = glmmTMB(Arachnid_abundance ~ Habitat + (1 | Site),
            ziformula = ~0,
            family = nbinom2(link = "log"),
            data = sega)
E = glmmTMB(Arachnid_abundance ~ elescale  + (1 | Site),
            ziformula = ~0,
            family = nbinom2(link = "log"),
            data = sega)
E2 = glmmTMB(Arachnid_abundance ~ I(elescale^2)  + (1 | Site),
             ziformula = ~0,
             family = nbinom2(link = "log"),
             data = sega)
EE2 = glmmTMB(Arachnid_abundance ~ elescale + I(elescale^2)  + (1 | Site),
              ziformula = ~0,
              family = nbinom2(link = "log"),
              data = sega)
EH = glmmTMB(Arachnid_abundance ~ elescale * Habitat   + (1 | Site),
             ziformula = ~0,
             family = nbinom2(link = "log"),
             data = sega)
E2H = glmmTMB(Arachnid_abundance ~ I(elescale^2) * Habitat   + (1 | Site),
              ziformula = ~0,
              family = nbinom2(link = "log"),
              data = sega)
EE2H = glmmTMB(Arachnid_abundance ~ elescale * Habitat + I(elescale^2)  + (1 | Site),
               ziformula = ~0,
               family = nbinom2(link = "log"),
               data = sega)
HD = glmmTMB(Arachnid_abundance ~ Habitat  + Season + (1 | Site),
             ziformula = ~0,
             family = nbinom2(link = "log"),
             data = sega)
ED = glmmTMB(Arachnid_abundance ~ elescale  + Season + (1 | Site),
             ziformula = ~0,
             family = nbinom2(link = "log"),
             data = sega)
E2D = glmmTMB(Arachnid_abundance ~ I(elescale^2) + Season + (1 | Site),
              ziformula = ~0,
              family = nbinom2(link = "log"),
              data = sega)
EE2D = glmmTMB(Arachnid_abundance ~ elescale + I(elescale^2) + Season + (1 | Site),
               ziformula = ~0,
               family = nbinom2(link = "log"),
               data = sega)
EHD = glmmTMB(Arachnid_abundance ~ elescale * Habitat  + Season + (1 | Site),
              ziformula = ~0,
              family = nbinom2(link = "log"),
              data = sega)
E2HD = glmmTMB(Arachnid_abundance ~ I(elescale^2) * Habitat  + Season + (1 | Site),
               ziformula = ~0,
               family = nbinom2(link = "log"),
               data = sega)
EE2HD = glmmTMB(Arachnid_abundance ~ elescale * Habitat + I(elescale^2) + Season + (1 | Site),
                ziformula = ~0,
                family = nbinom2(link = "log"),
                data = sega)
D = glmmTMB(Arachnid_abundance ~ Season + (1 | Site),
                ziformula = ~0,
                family = nbinom2(link = "log"),
                data = sega)
anova(N, H, E, E2, EE2, EH, E2H, EE2H, HD, ED, E2D, EE2D, EHD, E2HD, EE2HD, D)
```

```
#Best model statistics and predictions
summary(EE2HD)
```

```
##  Family: nbinom2  ( log )
## Formula:          
## Arachnid_abundance ~ elescale * Habitat + I(elescale^2) + Season +  
##     (1 | Site)
## Data: sega
## 
##      AIC      BIC   logLik deviance df.resid 
##    932.2    957.0   -458.1    916.2      155 
## 
## Random effects:
## 
## Conditional model:
##  Groups Name        Variance Std.Dev.
##  Site   (Intercept) 0.2138   0.4623  
## Number of obs: 163, groups:  Site, 20
## 
## Dispersion parameter for nbinom2 family (): 2.47 
## 
## Conditional model:
##                      Estimate Std. Error z value Pr(>|z|)    
## (Intercept)            2.0929     0.2261   9.255   <2e-16 ***
## elescale               0.2644     0.3132   0.844   0.3986    
## HabitatOpen           -0.2213     0.3007  -0.736   0.4619    
## I(elescale^2)         -0.3585     0.1677  -2.138   0.0325 *  
## SeasonMonsoon         -0.2808     0.1321  -2.126   0.0335 *  
## elescale:HabitatOpen  -0.9136     0.4488  -2.035   0.0418 *  
## ---
## Signif. codes:  0 '***' 0.001 '**' 0.01 '*' 0.05 '.' 0.1 ' ' 1
```

```
r.squaredGLMM(EE2HD)
```

```
## Warning in r.squaredGLMM.glmmTMB(EE2HD): the effects of zero-inflation and
## dispersion model are ignored
```

```
## Warning: the null model is correct only if all variables used by the original
## model remain unchanged.
```

```
##                 R2m       R2c
## delta     0.2152827 0.4316562
## lognormal 0.2438811 0.4889978
## trigamma  0.1812376 0.3633934
```

```
#Get average values of actual data for graph
sega2 <- sega %>%
  group_by(Season, Site, Habitat, Elevation) %>%
  summarise(Averages = mean(Arachnid_abundance))
```

```
## `summarise()` regrouping output by 'Season', 'Site', 'Habitat' (override with `.groups` argument)
```

```
# Make new dataframe for prediction:
newdat$beetle_pred <- predict(EE2H, newdata = newdat, type = "response", re.form=NULL)
preds <- predict(EE2H, newdat, se=T, allow.new.levels = T, type='response', re.form=~0)
newdat$pred = preds$fit
newdat$se = preds$se.fit
newdat$ulimit = newdat$pred + 1.96*newdat$se
newdat$llimit = newdat$pred - 1.96*newdat$se
#Plot Results, observed data as points, predicted values from model as trend lines
ggplot(data=newdat, aes(x = Elevation, y = pred))+
  geom_ribbon(aes(ymin=llimit, ymax=ulimit, fill = Habitat), alpha = 0.25)  +
  geom_point(aes(x = Elevation, y = Averages, color = Habitat), data=sega2) +
  geom_line(aes(color = Habitat), size=1) +
  labs(x = "Elevation (m)", y = "Arachnid Abundance") + 
  scale_color_manual(values=c("#228B22", "#FF8C00")) + scale_fill_manual(values=c("#FF8C00", "#228B22")) +
  theme(text = element_text(size = 20))
```

```
#Predator_richness
#Family test, poisson versus neg.binomial and zero-inflated 
Pmodel = glmmTMB(Predator_richness ~ 1  + (1 | Site),
                 ziformula = ~0,
                 family = poisson(link = "log"),
                 data = sega)
Pmodel_z = glmmTMB(Predator_richness ~ 1  + (1 | Site),
                   ziformula = ~1,
                   family = poisson(link = "log"),
                   data = sega)
Nmodel = glmmTMB(Predator_richness ~ 1  + (1 | Site),
                 ziformula = ~0,
                 family = nbinom2(link = "log"),
                 data = sega)
```

```
## Warning in fitTMB(TMBStruc): Model convergence problem; false convergence (8).
## See vignette('troubleshooting')
```

```
Nmodel_z = glmmTMB(Predator_richness ~ 1  + (1 | Site),
                   ziformula = ~1,
                   family = nbinom2(link = "log"),
                   data = sega)
anova(Pmodel, Pmodel_z, Nmodel, Nmodel_z)
```

```
#Find Best Model
N = glmmTMB(Predator_richness ~ 1  + (1 | Site),
            ziformula = ~0,
            family = poisson(link = "log"),
            data = sega)
H = glmmTMB(Predator_richness ~ Habitat  + (1 | Site),
            ziformula = ~0,
            family = poisson(link = "log"),
            data = sega)
E = glmmTMB(Predator_richness ~ elescale + (1 | Site),
            ziformula = ~0,
            family = poisson(link = "log"),
            data = sega)
E2 = glmmTMB(Predator_richness ~ I(elescale^2)  + (1 | Site),
             ziformula = ~0,
             family = poisson(link = "log"),
             data = sega)
EE2 = glmmTMB(Predator_richness ~ elescale + I(elescale^2)  + (1 | Site),
              ziformula = ~0,
              family = poisson(link = "log"),
              data = sega)
EH = glmmTMB(Predator_richness ~ elescale * Habitat   + (1 | Site),
             ziformula = ~0,
             family = poisson(link = "log"),
             data = sega)
E2H = glmmTMB(Predator_richness ~ I(elescale^2) * Habitat   + (1 | Site),
              ziformula = ~0,
              family = poisson(link = "log"),
              data = sega)
EE2H = glmmTMB(Predator_richness ~ elescale * Habitat + I(elescale^2)  + (1 | Site),
               ziformula = ~0,
               family = poisson(link = "log"),
               data = sega)
HD = glmmTMB(Predator_richness ~ Habitat  + Season + (1 | Site),
             ziformula = ~0,
             family = poisson(link = "log"),
             data = sega)
ED = glmmTMB(Predator_richness ~ elescale  + Season + (1 | Site),
             ziformula = ~0,
             family = poisson(link = "log"),
             data = sega)
E2D = glmmTMB(Predator_richness ~ I(elescale^2) + Season + (1 | Site),
              ziformula = ~0,
              family = poisson(link = "log"),
              data = sega)
EE2D = glmmTMB(Predator_richness ~ elescale + I(elescale^2) + Season + (1 | Site),
               ziformula = ~0,
               family = poisson(link = "log"),
               data = sega)
EHD = glmmTMB(Predator_richness ~ elescale * Habitat  + Season + (1 | Site),
              ziformula = ~0,
              family = poisson(link = "log"),
              data = sega)
E2HD = glmmTMB(Predator_richness ~ I(elescale^2) * Habitat  + Season + (1 | Site),
               ziformula = ~0,
               family = poisson(link = "log"),
               data = sega)
EE2HD = glmmTMB(Predator_richness ~ elescale * Habitat + I(elescale^2) + Season + (1 | Site),
                ziformula = ~0,
                family = poisson(link = "log"),
                data = sega)
D = glmmTMB(Predator_richness ~ Season + (1 | Site),
                ziformula = ~0,
                family = poisson(link = "log"),
                data = sega)
anova(N, H, D, E, E2, EE2, EH, E2H, EE2H, HD, ED, E2D, EE2D, EHD, E2HD, EE2HD)
```

```
#Best model statistics and predictions
summary(EE2H)
```

```
##  Family: poisson  ( log )
## Formula:          
## Predator_richness ~ elescale * Habitat + I(elescale^2) + (1 |      Site)
## Data: sega
## 
##      AIC      BIC   logLik deviance df.resid 
##    602.6    621.2   -295.3    590.6      157 
## 
## Random effects:
## 
## Conditional model:
##  Groups Name        Variance  Std.Dev. 
##  Site   (Intercept) 6.768e-10 2.602e-05
## Number of obs: 163, groups:  Site, 20
## 
## Conditional model:
##                      Estimate Std. Error z value Pr(>|z|)    
## (Intercept)           0.98934    0.08738  11.323  < 2e-16 ***
## elescale              0.19066    0.13385   1.424  0.15431    
## HabitatOpen           0.01146    0.11997   0.095  0.92393    
## I(elescale^2)        -0.12900    0.07157  -1.803  0.07147 .  
## elescale:HabitatOpen -0.58194    0.20500  -2.839  0.00453 ** 
## ---
## Signif. codes:  0 '***' 0.001 '**' 0.01 '*' 0.05 '.' 0.1 ' ' 1
```

```
r.squaredGLMM(EE2H)
```

```
## Warning in r.squaredGLMM.glmmTMB(EE2H): the effects of zero-inflation and
## dispersion model are ignored
```

```
## Warning: the null model is correct only if all variables used by the original
## model remain unchanged.
```

```
##                 R2m       R2c
## delta     0.1348968 0.1348968
## lognormal 0.1545607 0.1545607
## trigamma  0.1147203 0.1147203
```

```
#Get average values of actual data for graph
sega2 <- sega %>%
  group_by(Season, Site, Habitat, Elevation) %>%
  summarise(Averages = mean(Predator_richness))
```

```
## `summarise()` regrouping output by 'Season', 'Site', 'Habitat' (override with `.groups` argument)
```

```
# Make new dataframe for prediction:
newdat$beetle_pred <- predict(EE2H, newdata = newdat, type = "response", re.form=NULL)
preds <- predict(EE2H, newdat, se=T, allow.new.levels = T, type='response', re.form=~0)
newdat$pred = preds$fit
newdat$se = preds$se.fit
newdat$ulimit = newdat$pred + 1.96*newdat$se
newdat$llimit = newdat$pred - 1.96*newdat$se
#Plot Results, observed data as points, predicted values from model as trend lines
ggplot(data=newdat, aes(x = Elevation, y = pred))+
  geom_ribbon(aes(ymin=llimit, ymax=ulimit, fill = Habitat), alpha = 0.25)  +
  geom_point(aes(x = Elevation, y = Averages, color = Habitat), data=sega2) +
  geom_line(aes(color = Habitat), size=1) +
  labs(x = "Elevation (m)", y = "Predator Richness") + 
  scale_color_manual(values=c("#228B22", "#FF8C00")) + scale_fill_manual(values=c("#FF8C00", "#228B22")) +
  theme(text = element_text(size = 20))
```

```
#Predator_abundance
#Family test, poisson versus neg.binomial and zero-inflated 
Pmodel = glmmTMB(Predator_abundance ~ 1  + (1 | Site),
                 ziformula = ~0,
                 family = poisson(link = "log"),
                 data = sega)
Pmodel_z = glmmTMB(Predator_abundance ~ 1  + (1 | Site),
                   ziformula = ~1,
                   family = poisson(link = "log"),
                   data = sega)
Nmodel = glmmTMB(Predator_abundance ~ 1  + (1 | Site),
                 ziformula = ~0,
                 family = nbinom2(link = "log"),
                 data = sega)
Nmodel_z = glmmTMB(Predator_abundance ~ 1  + (1 | Site),
                   ziformula = ~1,
                   family = nbinom2(link = "log"),
                   data = sega)
anova(Pmodel, Pmodel_z, Nmodel, Nmodel_z)
```

```
#Find Best Model
N = glmmTMB(Predator_abundance ~ 1  + (1 | Site),
            ziformula = ~1,
            family = nbinom2(link = "log"),
            data = sega)
H = glmmTMB(Predator_abundance ~ Habitat  + (1 | Site),
            ziformula = ~1,
            family = nbinom2(link = "log"),
            data = sega)
E = glmmTMB(Predator_abundance ~ elescale   + (1 | Site),
            ziformula = ~1,
            family = nbinom2(link = "log"),
            data = sega)
E2 = glmmTMB(Predator_abundance ~ I(elescale^2)  + (1 | Site),
             ziformula = ~1,
             family = nbinom2(link = "log"),
             data = sega)
EE2 = glmmTMB(Predator_abundance ~ elescale + I(elescale^2)  + (1 | Site),
              ziformula = ~1,
              family = nbinom2(link = "log"),
              data = sega)
EH = glmmTMB(Predator_abundance ~ elescale * Habitat   + (1 | Site),
             ziformula = ~1,
             family = nbinom2(link = "log"),
             data = sega)
E2H = glmmTMB(Predator_abundance ~ I(elescale^2) * Habitat   + (1 | Site),
              ziformula = ~1,
              family = nbinom2(link = "log"),
              data = sega)
EE2H = glmmTMB(Predator_abundance ~ elescale * Habitat + I(elescale^2) + (1 | Site),
               ziformula = ~1,
               family = nbinom2(link = "log"),
               data = sega)
HD = glmmTMB(Predator_abundance ~ Habitat  + Season + (1 | Site),
             ziformula = ~1,
             family = nbinom2(link = "log"),
             data = sega)
ED = glmmTMB(Predator_abundance ~ elescale  + Season + (1 | Site),
             ziformula = ~1,
             family = nbinom2(link = "log"),
             data = sega)
E2D = glmmTMB(Predator_abundance ~ I(elescale^2) + Season + (1 | Site),
              ziformula = ~1,
              family = nbinom2(link = "log"),
              data = sega)
EE2D = glmmTMB(Predator_abundance ~ elescale + I(elescale^2) + Season + (1 | Site),
               ziformula = ~1,
               family = nbinom2(link = "log"),
               data = sega)
EHD = glmmTMB(Predator_abundance ~ elescale * Habitat  + Season + (1 | Site),
              ziformula = ~1,
              family = nbinom2(link = "log"),
              data = sega)
E2HD = glmmTMB(Predator_abundance ~ I(elescale^2) * Habitat  + Season + (1 | Site),
               ziformula = ~1,
               family = nbinom2(link = "log"),
               data = sega)
EE2HD = glmmTMB(Predator_abundance ~ elescale * Habitat + I(elescale^2) + Season + (1 | Site),
                ziformula = ~1,
                family = nbinom2(link = "log"),
                data = sega)
D = glmmTMB(Predator_abundance ~ Season + (1 | Site),
                ziformula = ~1,
                family = nbinom2(link = "log"),
                data = sega)
anova(N, H, D, E, E2, EE2, EH, E2H, EE2H, HD, ED, E2D, EE2D, EHD, E2HD, EE2HD)
```

```
#Best model statistics and predictions
summary(EE2H)
```

```
##  Family: nbinom2  ( log )
## Formula:          
## Predator_abundance ~ elescale * Habitat + I(elescale^2) + (1 |      Site)
## Zero inflation:                      ~1
## Data: sega
## 
##      AIC      BIC   logLik deviance df.resid 
##    991.5   1016.3   -487.8    975.5      155 
## 
## Random effects:
## 
## Conditional model:
##  Groups Name        Variance Std.Dev.
##  Site   (Intercept) 0.332    0.5762  
## Number of obs: 163, groups:  Site, 20
## 
## Dispersion parameter for nbinom2 family (): 2.56 
## 
## Conditional model:
##                      Estimate Std. Error z value Pr(>|z|)    
## (Intercept)           1.75234    0.26525   6.606 3.94e-11 ***
## elescale              0.74877    0.36274   2.064  0.03900 *  
## HabitatOpen           0.09082    0.35816   0.254  0.79982    
## I(elescale^2)        -0.30032    0.18855  -1.593  0.11121    
## elescale:HabitatOpen -1.44936    0.50457  -2.872  0.00407 ** 
## ---
## Signif. codes:  0 '***' 0.001 '**' 0.01 '*' 0.05 '.' 0.1 ' ' 1
## 
## Zero-inflation model:
##             Estimate Std. Error z value Pr(>|z|)    
## (Intercept)   -4.487      1.110  -4.041 5.31e-05 ***
## ---
## Signif. codes:  0 '***' 0.001 '**' 0.01 '*' 0.05 '.' 0.1 ' ' 1
```

```
r.squaredGLMM(EE2H)
```

```
## Warning in r.squaredGLMM.glmmTMB(EE2H): the effects of zero-inflation and
## dispersion model are ignored
```

```
## Warning: the null model is correct only if all variables used by the original
## model remain unchanged.
```

```
##                 R2m       R2c
## delta     0.1994166 0.5132345
## lognormal 0.2201348 0.5665566
## trigamma  0.1740321 0.4479030
```

```
#Get average values of actual data for graph
sega2 <- sega %>%
  group_by(Season, Site, Habitat, Elevation) %>%
  summarise(Averages = mean(Predator_abundance))
```

```
## `summarise()` regrouping output by 'Season', 'Site', 'Habitat' (override with `.groups` argument)
```

```
# Make new dataframe for prediction:
newdat$beetle_pred <- predict(EE2H, newdata = newdat, type = "response", re.form=NULL)
preds <- predict(EE2H, newdat, se=T, allow.new.levels = T, type='response', re.form=~0)
newdat$pred = preds$fit
newdat$se = preds$se.fit
newdat$ulimit = newdat$pred + 1.96*newdat$se
newdat$llimit = newdat$pred - 1.96*newdat$se
#Plot Results, observed data as points, predicted values from model as trend lines
ggplot(data=newdat, aes(x = Elevation, y = pred))+
  geom_ribbon(aes(ymin=llimit, ymax=ulimit, fill = Habitat), alpha = 0.25)  +
  geom_point(aes(x = Elevation, y = Averages, color = Habitat), data=sega2) +
  geom_line(aes(color = Habitat), size=1) +
  labs(x = "Elevation (m)", y = "Predator Abundance") + 
  scale_color_manual(values=c("#228B22", "#FF8C00")) + scale_fill_manual(values=c("#FF8C00", "#228B22")) +
  theme(text = element_text(size = 20))
```

```
#Herbivore_richness
#Family test, poisson versus neg.binomial and zero-inflated 
Pmodel = glmmTMB(Herbivore_richness ~ 1  + (1 | Site),
                 ziformula = ~0,
                 family = poisson(link = "log"),
                 data = sega)
Pmodel_z = glmmTMB(Herbivore_richness ~ 1  + (1 | Site),
                   ziformula = ~1,
                   family = poisson(link = "log"),
                   data = sega)
Nmodel = glmmTMB(Herbivore_richness ~ 1  + (1 | Site),
                 ziformula = ~0,
                 family = nbinom2(link = "log"),
                 data = sega)
```

```
## Warning in fitTMB(TMBStruc): Model convergence problem; non-positive-definite
## Hessian matrix. See vignette('troubleshooting')
```

```
## Warning in fitTMB(TMBStruc): Model convergence problem; false convergence (8).
## See vignette('troubleshooting')
```

```
Nmodel_z = glmmTMB(Herbivore_richness ~ 1  + (1 | Site),
                   ziformula = ~1,
                   family = nbinom2(link = "log"),
                   data = sega)
```

```
## Warning in fitTMB(TMBStruc): Model convergence problem; false convergence (8).
## See vignette('troubleshooting')
```

```
anova(Pmodel, Pmodel_z, Nmodel, Nmodel_z)
```

```
#Find Best Model
N = glmmTMB(Herbivore_richness ~ 1  + (1 | Site),
            ziformula = ~0,
            family = poisson(link = "log"),
            data = sega)
H = glmmTMB(Herbivore_richness ~ Habitat  + (1 | Site),
            ziformula = ~0,
            family = poisson(link = "log"),
            data = sega)
E = glmmTMB(Herbivore_richness ~ elescale + (1 | Site),
            ziformula = ~0,
            family = poisson(link = "log"),
            data = sega)
E2 = glmmTMB(Herbivore_richness ~ I(elescale^2)  + (1 | Site),
             ziformula = ~0,
             family = poisson(link = "log"),
             data = sega)
EE2 = glmmTMB(Herbivore_richness ~ elescale + I(elescale^2)  + (1 | Site),
              ziformula = ~0,
              family = poisson(link = "log"),
              data = sega)
EH = glmmTMB(Herbivore_richness ~ elescale * Habitat   + (1 | Site),
             ziformula = ~0,
             family = poisson(link = "log"),
             data = sega)
E2H = glmmTMB(Herbivore_richness ~ I(elescale^2) * Habitat   + (1 | Site),
              ziformula = ~0,
              family = poisson(link = "log"),
              data = sega)
EE2H = glmmTMB(Herbivore_richness ~ elescale * Habitat + I(elescale^2)  + (1 | Site),
               ziformula = ~0,
               family = poisson(link = "log"),
               data = sega)
HD = glmmTMB(Herbivore_richness ~ Habitat  + Season + (1 | Site),
             ziformula = ~0,
             family = poisson(link = "log"),
             data = sega)
ED = glmmTMB(Herbivore_richness ~ elescale  + Season + (1 | Site),
             ziformula = ~0,
             family = poisson(link = "log"),
             data = sega)
E2D = glmmTMB(Herbivore_richness ~ I(elescale^2) + Season + (1 | Site),
              ziformula = ~0,
              family = poisson(link = "log"),
              data = sega)
EE2D = glmmTMB(Herbivore_richness ~ elescale + I(elescale^2) + Season + (1 | Site),
               ziformula = ~0,
               family = poisson(link = "log"),
               data = sega)
EHD = glmmTMB(Herbivore_richness ~ elescale * Habitat  + Season + (1 | Site),
              ziformula = ~0,
              family = poisson(link = "log"),
              data = sega)
E2HD = glmmTMB(Herbivore_richness ~ I(elescale^2) * Habitat  + Season + (1 | Site),
               ziformula = ~0,
               family = poisson(link = "log"),
               data = sega)
EE2HD = glmmTMB(Herbivore_richness ~ elescale * Habitat + I(elescale^2) + Season + (1 | Site),
                ziformula = ~0,
                family = poisson(link = "log"),
                data = sega)
D = glmmTMB(Herbivore_richness ~ Season + (1 | Site),
                ziformula = ~0,
                family = poisson(link = "log"),
                data = sega)
anova(N, H, E, E2, EE2, EH, E2H, EE2H, HD, ED, E2D, EE2D, EHD, E2HD, EE2HD, D)
```

```
#Best model statistics and predictions
summary(H)
```

```
##  Family: poisson  ( log )
## Formula:          Herbivore_richness ~ Habitat + (1 | Site)
## Data: sega
## 
##      AIC      BIC   logLik deviance df.resid 
##    431.8    441.1   -212.9    425.8      160 
## 
## Random effects:
## 
## Conditional model:
##  Groups Name        Variance Std.Dev.
##  Site   (Intercept) 0.09001  0.3     
## Number of obs: 163, groups:  Site, 20
## 
## Conditional model:
##             Estimate Std. Error z value Pr(>|z|)  
## (Intercept)  -0.2217     0.1652  -1.342   0.1796  
## HabitatOpen   0.5182     0.2094   2.475   0.0133 *
## ---
## Signif. codes:  0 '***' 0.001 '**' 0.01 '*' 0.05 '.' 0.1 ' ' 1
```

```
r.squaredGLMM(H)
```

```
## Warning in r.squaredGLMM.glmmTMB(H): the effects of zero-inflation and
## dispersion model are ignored
```

```
## Warning: the null model is correct only if all variables used by the original
## model remain unchanged.
```

```
##                  R2m       R2c
## delta     0.06516471 0.1527393
## lognormal 0.08549926 0.2004014
## trigamma  0.04449385 0.1042890
```

```
#Get average values of actual data for graph
sega2 <- sega %>%
  group_by(Season, Site, Habitat, Elevation) %>%
  summarise(Averages = mean(Herbivore_richness))
```

```
## `summarise()` regrouping output by 'Season', 'Site', 'Habitat' (override with `.groups` argument)
```

```
# Make new dataframe for prediction:
newdat$beetle_pred <- predict(H, newdata = newdat, type = "response", re.form=NULL)
preds <- predict(H, newdat, se=T, allow.new.levels = T, type='response', re.form=~0)
newdat$pred = preds$fit
newdat$se = preds$se.fit
newdat$ulimit = newdat$pred + 1.96*newdat$se
newdat$llimit = newdat$pred - 1.96*newdat$se
#Plot Results, observed data as points, predicted values from model as trend lines
ggplot(data=newdat, aes(x = Elevation, y = pred))+
  geom_ribbon(aes(ymin=llimit, ymax=ulimit, fill = Habitat), alpha = 0.25)  +
  geom_point(aes(x = Elevation, y = Averages, color = Habitat), data=sega2) +
  geom_line(aes(color = Habitat), size=1) +
  labs(x = "Elevation (m)", y = "Herbivore Richness") + 
  scale_color_manual(values=c("#228B22", "#FF8C00")) + scale_fill_manual(values=c("#FF8C00", "#228B22")) +
  theme(text = element_text(size = 20))
```

```
#Herbivore_abundance
#Family test, poisson versus neg.binomial and zero-inflated 
Pmodel = glmmTMB(Herbivore_abundance ~ 1  + (1 | Site),
                 ziformula = ~0,
                 family = poisson(link = "log"),
                 data = sega)
Pmodel_z = glmmTMB(Herbivore_abundance ~ 1  + (1 | Site),
                   ziformula = ~1,
                   family = poisson(link = "log"),
                   data = sega)
Nmodel = glmmTMB(Herbivore_abundance ~ 1  + (1 | Site),
                 ziformula = ~0,
                 family = nbinom2(link = "log"),
                 data = sega)
Nmodel_z = glmmTMB(Herbivore_abundance ~ 1  + (1 | Site),
                   ziformula = ~1,
                   family = nbinom2(link = "log"),
                   data = sega)
anova(Pmodel, Pmodel_z, Nmodel, Nmodel_z)
```

```
#Find Best Model
N = glmmTMB(Herbivore_abundance ~ 1  + (1 | Site),
            ziformula = ~1,
            family = nbinom2(link = "log"),
            data = sega)
H = glmmTMB(Herbivore_abundance ~ Habitat  + (1 | Site),
            ziformula = ~1,
            family = nbinom2(link = "log"),
            data = sega)
E = glmmTMB(Herbivore_abundance ~ elescale   + (1 | Site),
            ziformula = ~1,
            family = nbinom2(link = "log"),
            data = sega)
E2 = glmmTMB(Herbivore_abundance ~ I(elescale^2)  + (1 | Site),
             ziformula = ~1,
             family = nbinom2(link = "log"),
             data = sega)
EE2 = glmmTMB(Herbivore_abundance ~ elescale + I(elescale^2)  + (1 | Site),
              ziformula = ~1,
              family = nbinom2(link = "log"),
              data = sega)
EH = glmmTMB(Herbivore_abundance ~ elescale * Habitat   + (1 | Site),
             ziformula = ~1,
             family = nbinom2(link = "log"),
             data = sega)
E2H = glmmTMB(Herbivore_abundance ~ I(elescale^2) * Habitat   + (1 | Site),
              ziformula = ~1,
              family = nbinom2(link = "log"),
              data = sega)
EE2H = glmmTMB(Herbivore_abundance ~ elescale * Habitat + I(elescale^2) + (1 | Site),
               ziformula = ~1,
               family = nbinom2(link = "log"),
               data = sega)
HD = glmmTMB(Herbivore_abundance ~ Habitat  + Season + (1 | Site),
             ziformula = ~1,
             family = nbinom2(link = "log"),
             data = sega)
ED = glmmTMB(Herbivore_abundance ~ elescale  + Season + (1 | Site),
             ziformula = ~1,
             family = nbinom2(link = "log"),
             data = sega)
E2D = glmmTMB(Herbivore_abundance ~ I(elescale^2) + Season + (1 | Site),
              ziformula = ~1,
              family = nbinom2(link = "log"),
              data = sega)
EE2D = glmmTMB(Herbivore_abundance ~ elescale + I(elescale^2) + Season + (1 | Site),
               ziformula = ~1,
               family = nbinom2(link = "log"),
               data = sega)
EHD = glmmTMB(Herbivore_abundance ~ elescale * Habitat  + Season + (1 | Site),
              ziformula = ~1,
              family = nbinom2(link = "log"),
              data = sega)
E2HD = glmmTMB(Herbivore_abundance ~ I(elescale^2) * Habitat  + Season + (1 | Site),
               ziformula = ~1,
               family = nbinom2(link = "log"),
               data = sega)
EE2HD = glmmTMB(Herbivore_abundance ~ elescale * Habitat + I(elescale^2) + Season + (1 | Site),
                ziformula = ~1,
                family = nbinom2(link = "log"),
                data = sega)
D = glmmTMB(Herbivore_abundance ~ Season + (1 | Site),
                ziformula = ~1,
                family = nbinom2(link = "log"),
                data = sega)
anova(N, H, E, E2, EE2, EH, E2H, EE2H, HD, ED, E2D, EE2D, EHD, E2HD, EE2HD, D)
```

```
#Best model statistics and predictions
summary(E)
```

```
##  Family: nbinom2  ( log )
## Formula:          Herbivore_abundance ~ elescale + (1 | Site)
## Zero inflation:                       ~1
## Data: sega
## 
##      AIC      BIC   logLik deviance df.resid 
##    757.4    772.8   -373.7    747.4      158 
## 
## Random effects:
## 
## Conditional model:
##  Groups Name        Variance Std.Dev.
##  Site   (Intercept) 0.8621   0.9285  
## Number of obs: 163, groups:  Site, 20
## 
## Dispersion parameter for nbinom2 family (): 0.664 
## 
## Conditional model:
##             Estimate Std. Error z value Pr(>|z|)    
## (Intercept)   0.9622     0.2418   3.979 6.93e-05 ***
## elescale      0.3372     0.2323   1.452    0.147    
## ---
## Signif. codes:  0 '***' 0.001 '**' 0.01 '*' 0.05 '.' 0.1 ' ' 1
## 
## Zero-inflation model:
##             Estimate Std. Error z value Pr(>|z|)
## (Intercept)   -18.42    4610.40  -0.004    0.997
```

```
r.squaredGLMM(E)
```

```
## Warning in r.squaredGLMM.glmmTMB(E): the effects of zero-inflation and
## dispersion model are ignored
```

```
## Warning: the null model is correct only if all variables used by the original
## model remain unchanged.
```

```
##                  R2m       R2c
## delta     0.04193881 0.3598258
## lognormal 0.05737349 0.4922519
## trigamma  0.02337584 0.2005596
```

```
#Get average values of actual data for graph
sega2 <- sega %>%
  group_by(Season, Site, Habitat, Elevation) %>%
  summarise(Averages = mean(Herbivore_abundance))
```

```
## `summarise()` regrouping output by 'Season', 'Site', 'Habitat' (override with `.groups` argument)
```

```
# Make new dataframe for prediction:
newdat$beetle_pred <- predict(E, newdata = newdat, type = "response", re.form=NULL)
preds <- predict(E, newdat, se=T, allow.new.levels = T, type='response', re.form=~0)
newdat$pred = preds$fit
newdat$se = preds$se.fit
newdat$ulimit = newdat$pred + 1.96*newdat$se
newdat$llimit = newdat$pred - 1.96*newdat$se
#Plot Results, observed data as points, predicted values from model as trend lines
ggplot(data=newdat, aes(x = Elevation, y = pred))+
  geom_ribbon(aes(ymin=llimit, ymax=ulimit, fill = Habitat), alpha = 0.25)  +
  geom_point(aes(x = Elevation, y = Averages, color = Habitat), data=sega2) +
  geom_line(aes(color = Habitat), size=1) +
  labs(x = "Elevation (m)", y = "Herbivore Abundance") + 
  scale_color_manual(values=c("#228B22", "#FF8C00")) + scale_fill_manual(values=c("#FF8C00", "#228B22")) +
  theme(text = element_text(size = 20))
```

```
#Detritivore_Richness
#Family test, poisson versus neg.binomial and zero-inflated 
Pmodel = glmmTMB(Detritivore_Richness ~ 1  + (1 | Site),
                 ziformula = ~0,
                 family = poisson(link = "log"),
                 data = sega)
Pmodel_z = glmmTMB(Detritivore_Richness ~ 1  + (1 | Site),
                   ziformula = ~1,
                   family = poisson(link = "log"),
                   data = sega)
Nmodel = glmmTMB(Detritivore_Richness ~ 1  + (1 | Site),
                 ziformula = ~0,
                 family = nbinom2(link = "log"),
                 data = sega)
```

```
## Warning in fitTMB(TMBStruc): Model convergence problem; false convergence (8).
## See vignette('troubleshooting')
```

```
Nmodel_z = glmmTMB(Detritivore_Richness ~ 1  + (1 | Site),
                   ziformula = ~1,
                   family = nbinom2(link = "log"),
                   data = sega)
```

```
## Warning in fitTMB(TMBStruc): Model convergence problem; false convergence (8).
## See vignette('troubleshooting')
```

```
anova(Pmodel, Pmodel_z, Nmodel, Nmodel_z)
```

```
#Find Best Model
N = glmmTMB(Detritivore_Richness ~ 1  + (1 | Site),
            ziformula = ~0,
            family = poisson(link = "log"),
            data = sega)
H = glmmTMB(Detritivore_Richness ~ Habitat  + (1 | Site),
            ziformula = ~0,
            family = poisson(link = "log"),
            data = sega)
E = glmmTMB(Detritivore_Richness ~ elescale + (1 | Site),
            ziformula = ~0,
            family = poisson(link = "log"),
            data = sega)
E2 = glmmTMB(Detritivore_Richness ~ I(elescale^2)  + (1 | Site),
             ziformula = ~0,
             family = poisson(link = "log"),
             data = sega)
EE2 = glmmTMB(Detritivore_Richness ~ elescale + I(elescale^2)  + (1 | Site),
              ziformula = ~0,
              family = poisson(link = "log"),
              data = sega)
EH = glmmTMB(Detritivore_Richness ~ elescale * Habitat   + (1 | Site),
             ziformula = ~0,
             family = poisson(link = "log"),
             data = sega)
E2H = glmmTMB(Detritivore_Richness ~ I(elescale^2) * Habitat   + (1 | Site),
              ziformula = ~0,
              family = poisson(link = "log"),
              data = sega)
EE2H = glmmTMB(Detritivore_Richness ~ elescale * Habitat + I(elescale^2)  + (1 | Site),
               ziformula = ~0,
               family = poisson(link = "log"),
               data = sega)
HD = glmmTMB(Detritivore_Richness ~ Habitat  + Season + (1 | Site),
             ziformula = ~0,
             family = poisson(link = "log"),
             data = sega)
ED = glmmTMB(Detritivore_Richness ~ elescale  + Season + (1 | Site),
             ziformula = ~0,
             family = poisson(link = "log"),
             data = sega)
E2D = glmmTMB(Detritivore_Richness ~ I(elescale^2) + Season + (1 | Site),
              ziformula = ~0,
              family = poisson(link = "log"),
              data = sega)
EE2D = glmmTMB(Detritivore_Richness ~ elescale + I(elescale^2) + Season + (1 | Site),
               ziformula = ~0,
               family = poisson(link = "log"),
               data = sega)
EHD = glmmTMB(Detritivore_Richness ~ elescale * Habitat  + Season + (1 | Site),
              ziformula = ~0,
              family = poisson(link = "log"),
              data = sega)
E2HD = glmmTMB(Detritivore_Richness ~ I(elescale^2) * Habitat  + Season + (1 | Site),
               ziformula = ~0,
               family = poisson(link = "log"),
               data = sega)
EE2HD = glmmTMB(Detritivore_Richness ~ elescale * Habitat + I(elescale^2) + Season + (1 | Site),
                ziformula = ~0,
                family = poisson(link = "log"),
                data = sega)
D = glmmTMB(Detritivore_Richness ~ Season + (1 | Site),
                ziformula = ~0,
                family = poisson(link = "log"),
                data = sega)
anova(N, H, E, E2, EE2, EH, E2H, EE2H, HD, ED, E2D, EE2D, EHD, E2HD, EE2HD, D)
```

```
#Best model statistics and predictions
summary(H)
```

```
##  Family: poisson  ( log )
## Formula:          Detritivore_Richness ~ Habitat + (1 | Site)
## Data: sega
## 
##      AIC      BIC   logLik deviance df.resid 
##    678.9    688.2   -336.4    672.9      160 
## 
## Random effects:
## 
## Conditional model:
##  Groups Name        Variance Std.Dev.
##  Site   (Intercept) 0.08454  0.2908  
## Number of obs: 163, groups:  Site, 20
## 
## Conditional model:
##             Estimate Std. Error z value Pr(>|z|)    
## (Intercept)   1.5395     0.1117  13.777   <2e-16 ***
## HabitatOpen  -0.2071     0.1535  -1.349    0.177    
## ---
## Signif. codes:  0 '***' 0.001 '**' 0.01 '*' 0.05 '.' 0.1 ' ' 1
```

```
r.squaredGLMM(H)
```

```
## Warning in r.squaredGLMM.glmmTMB(H): the effects of zero-inflation and
## dispersion model are ignored
```

```
## Warning: the null model is correct only if all variables used by the original
## model remain unchanged.
```

```
##                  R2m       R2c
## delta     0.03294319 0.2933848
## lognormal 0.03543988 0.3156198
## trigamma  0.03030178 0.2698610
```

```
#Get average values of actual data for graph
sega2 <- sega %>%
  group_by(Season, Site, Habitat, Elevation) %>%
  summarise(Averages = mean(Detritivore_Richness))
```

```
## `summarise()` regrouping output by 'Season', 'Site', 'Habitat' (override with `.groups` argument)
```

```
# Make new dataframe for prediction:
newdat$beetle_pred <- predict(H, newdata = newdat, type = "response", re.form=NULL)
preds <- predict(H, newdat, se=T, allow.new.levels = T, type='response', re.form=~0)
newdat$pred = preds$fit
newdat$se = preds$se.fit
newdat$ulimit = newdat$pred + 1.96*newdat$se
newdat$llimit = newdat$pred - 1.96*newdat$se
#Plot Results, observed data as points, predicted values from model as trend lines
ggplot(data=newdat, aes(x = Elevation, y = pred))+
  geom_ribbon(aes(ymin=llimit, ymax=ulimit, fill = Habitat), alpha = 0.25)  +
  geom_point(aes(x = Elevation, y = Averages, color = Habitat), data=sega2) +
  geom_line(aes(color = Habitat), size=1) +
  labs(x = "Elevation (m)", y = "Detritivore Richness") + 
  scale_color_manual(values=c("#228B22", "#FF8C00")) + scale_fill_manual(values=c("#FF8C00", "#228B22")) +
  theme(text = element_text(size = 20))
```

```
#Detritivore_Abundance
#Family test, poisson versus neg.binomial and zero-inflated 
Pmodel = glmmTMB(Detritivore_Abundance ~ 1  + (1 | Site),
                 ziformula = ~0,
                 family = poisson(link = "log"),
                 data = sega)
Pmodel_z = glmmTMB(Detritivore_Abundance ~ 1  + (1 | Site),
                   ziformula = ~1,
                   family = poisson(link = "log"),
                   data = sega)
Nmodel = glmmTMB(Detritivore_Abundance ~ 1  + (1 | Site),
                 ziformula = ~0,
                 family = nbinom2(link = "log"),
                 data = sega)
Nmodel_z = glmmTMB(Detritivore_Abundance ~ 1  + (1 | Site),
                   ziformula = ~1,
                   family = nbinom2(link = "log"),
                   data = sega)
anova(Pmodel, Pmodel_z, Nmodel, Nmodel_z)
```

```
#Find Best Model
N = glmmTMB(Detritivore_Abundance ~ 1  + (1 | Site),
            ziformula = ~1,
            family = nbinom2(link = "log"),
            data = sega)
H = glmmTMB(Detritivore_Abundance ~ Habitat  + (1 | Site),
            ziformula = ~1,
            family = nbinom2(link = "log"),
            data = sega)
E = glmmTMB(Detritivore_Abundance ~ elescale   + (1 | Site),
            ziformula = ~1,
            family = nbinom2(link = "log"),
            data = sega)
E2 = glmmTMB(Detritivore_Abundance ~ I(elescale^2)  + (1 | Site),
             ziformula = ~1,
             family = nbinom2(link = "log"),
             data = sega)
EE2 = glmmTMB(Detritivore_Abundance ~ elescale + I(elescale^2)  + (1 | Site),
              ziformula = ~1,
              family = nbinom2(link = "log"),
              data = sega)
EH = glmmTMB(Detritivore_Abundance ~ elescale * Habitat   + (1 | Site),
             ziformula = ~1,
             family = nbinom2(link = "log"),
             data = sega)
E2H = glmmTMB(Detritivore_Abundance ~ I(elescale^2) * Habitat   + (1 | Site),
              ziformula = ~1,
              family = nbinom2(link = "log"),
              data = sega)
EE2H = glmmTMB(Detritivore_Abundance ~ elescale * Habitat + I(elescale^2) + (1 | Site),
               ziformula = ~1,
               family = nbinom2(link = "log"),
               data = sega)
HD = glmmTMB(Detritivore_Abundance ~ Habitat  + Season + (1 | Site),
             ziformula = ~1,
             family = nbinom2(link = "log"),
             data = sega)
ED = glmmTMB(Detritivore_Abundance ~ elescale  + Season + (1 | Site),
             ziformula = ~1,
             family = nbinom2(link = "log"),
             data = sega)
E2D = glmmTMB(Detritivore_Abundance ~ I(elescale^2) + Season + (1 | Site),
              ziformula = ~1,
              family = nbinom2(link = "log"),
              data = sega)
EE2D = glmmTMB(Detritivore_Abundance ~ elescale + I(elescale^2) + Season + (1 | Site),
               ziformula = ~1,
               family = nbinom2(link = "log"),
               data = sega)
EHD = glmmTMB(Detritivore_Abundance ~ elescale * Habitat  + Season + (1 | Site),
              ziformula = ~1,
              family = nbinom2(link = "log"),
              data = sega)
E2HD = glmmTMB(Detritivore_Abundance ~ I(elescale^2) * Habitat  + Season + (1 | Site),
               ziformula = ~1,
               family = nbinom2(link = "log"),
               data = sega)
EE2HD = glmmTMB(Detritivore_Abundance ~ elescale * Habitat + I(elescale^2) + Season + (1 | Site),
                ziformula = ~1,
                family = nbinom2(link = "log"),
                data = sega)
D = glmmTMB(Detritivore_Abundance ~ Season + (1 | Site),
                ziformula = ~1,
                family = nbinom2(link = "log"),
                data = sega)
anova(N, H, E, E2, EE2, EH, E2H, EE2H, HD, ED, E2D, EE2D, EHD, E2HD, EE2HD, D)
```

```
#Best model statistics and predictions
summary(N)
```

```
##  Family: nbinom2  ( log )
## Formula:          Detritivore_Abundance ~ 1 + (1 | Site)
## Zero inflation:                         ~1
## Data: sega
## 
##      AIC      BIC   logLik deviance df.resid 
##   1527.5   1539.9   -759.8   1519.5      159 
## 
## Random effects:
## 
## Conditional model:
##  Groups Name        Variance Std.Dev.
##  Site   (Intercept) 0.7263   0.8523  
## Number of obs: 163, groups:  Site, 20
## 
## Dispersion parameter for nbinom2 family (): 1.56 
## 
## Conditional model:
##             Estimate Std. Error z value Pr(>|z|)    
## (Intercept)   3.4969     0.2029   17.23   <2e-16 ***
## ---
## Signif. codes:  0 '***' 0.001 '**' 0.01 '*' 0.05 '.' 0.1 ' ' 1
## 
## Zero-inflation model:
##             Estimate Std. Error z value Pr(>|z|)
## (Intercept)   -21.38    3722.26  -0.006    0.995
```

```
r.squaredGLMM(N)
```

```
## Warning in r.squaredGLMM.glmmTMB(N): the effects of zero-inflation and
## dispersion model are ignored
```

```
## Warning: the null model is correct only if all variables used by the original
## model remain unchanged.
```

```
##           R2m       R2c
## delta       0 0.5237528
## lognormal   0 0.5888759
## trigamma    0 0.4403141
```

```
#Get average values of actual data for graph
sega2 <- sega %>%
  group_by(Season, Site, Habitat, Elevation) %>%
  summarise(Averages = mean(Detritivore_Abundance))
```

```
## `summarise()` regrouping output by 'Season', 'Site', 'Habitat' (override with `.groups` argument)
```

```
# Make new dataframe for prediction:
newdat$beetle_pred <- predict(E2, newdata = newdat, type = "response", re.form=NULL)
preds <- predict(E2, newdat, se=T, allow.new.levels = T, type='response', re.form=~0)
newdat$pred = preds$fit
newdat$se = preds$se.fit
newdat$ulimit = newdat$pred + 1.96*newdat$se
newdat$llimit = newdat$pred - 1.96*newdat$se
#Plot Results, observed data as points, predicted values from model as trend lines
ggplot(data=newdat, aes(x = Elevation, y = pred))+
  geom_ribbon(aes(ymin=llimit, ymax=ulimit, fill = Habitat), alpha = 0.25)  +
  geom_point(aes(x = Elevation, y = Averages, color = Habitat), data=sega2) +
  geom_line(aes(color = Habitat), size=1) +
  labs(x = "Elevation (m)", y = "Detritivore Abundance") + 
  scale_color_manual(values=c("#228B22", "#FF8C00")) + scale_fill_manual(values=c("#FF8C00", "#228B22")) +
  theme(text = element_text(size = 20))
```

```
#manyGLMs analysis for individual taxa
#Variables
Dat <- sega$Date
Hab <- sega$Habitat
Sit <- sega$Site
bugs <- mvabund(sega[,23:145])
#Find best model
modd <-manyglm(bugs~elescale)
sum(AIC(modd))
```

```
## [1] 11050.96
```

```
modd <-manyglm(bugs~elescale + Dat)
sum(AIC(modd))
```

```
## [1] 10962.32
```

```
modd <-manyglm(bugs~elescale * Hab + Dat)
sum(AIC(modd))
```

```
## [1] 10880.44
```

```
mode <-manyglm(bugs~elescale + I(elescale^2))
sum(AIC(mode))
```

```
## [1] 10771.11
```

```
modv <-manyglm(bugs~elescale + I(elescale^2) + Dat)
sum(AIC(modv))
```

```
## [1] 10684.99
```

```
modv <-manyglm(bugs~elescale * Hab + I(elescale^2) + Dat)
sum(AIC(modv))
```

```
## [1] 10624.04
```

```
#test dispersion
plot(modv)
```

```
#get model print-out
options(max.print=1000000)
#BUGZ <- anova(modv, p.uni="adjusted")  #takes over an hour to run!
```

```
#Ordination NMDS analysis
#make arthropod data matrices and habitat variables
bugs <- mvabund(sega[,23:145])
S <- sega$Site
LZ<-sega$LZ
LZH <-sega$LZH
#scaled Environmental Variables
Env <-sega[,194:204]
Env <-  scale(Env)
Env <-data.frame(Env)
Env
```

```
Elescale <-Env$Elevation
E2 <-Env$E2
Pscale <-Env$AvPrecip
Tscale <-Env$AvTemp
ord<-metaMDS(bugs, distance = "bray",k=2, try=100, trymax=100, autotransform=TRUE, expand=TRUE, plot=FALSE)
```

```
## Square root transformation
## Wisconsin double standardization
## Run 0 stress 0.2551202 
## Run 1 stress 0.2558745 
## Run 2 stress 0.2561436 
## Run 3 stress 0.2576192 
## Run 4 stress 0.2594724 
## Run 5 stress 0.2618278 
## Run 6 stress 0.2651706 
## Run 7 stress 0.2589166 
## Run 8 stress 0.2565994 
## Run 9 stress 0.2569923 
## Run 10 stress 0.2556574 
## Run 11 stress 0.2614829 
## Run 12 stress 0.2561937 
## Run 13 stress 0.261586 
## Run 14 stress 0.2552976 
## ... Procrustes: rmse 0.01777801  max resid 0.1271517 
## Run 15 stress 0.2611861 
## Run 16 stress 0.2554827 
## ... Procrustes: rmse 0.01708107  max resid 0.1257842 
## Run 17 stress 0.2577764 
## Run 18 stress 0.2577186 
## Run 19 stress 0.2566542 
## Run 20 stress 0.2552572 
## ... Procrustes: rmse 0.01870473  max resid 0.128041 
## Run 21 stress 0.2568563 
## Run 22 stress 0.2602318 
## Run 23 stress 0.2627651 
## Run 24 stress 0.2590888 
## Run 25 stress 0.2610651 
## Run 26 stress 0.2563758 
## Run 27 stress 0.2629777 
## Run 28 stress 0.2599846 
## Run 29 stress 0.2568074 
## Run 30 stress 0.256592 
## Run 31 stress 0.2657808 
## Run 32 stress 0.2705957 
## Run 33 stress 0.2607113 
## Run 34 stress 0.2581984 
## Run 35 stress 0.2600922 
## Run 36 stress 0.2586704 
## Run 37 stress 0.2583336 
## Run 38 stress 0.2598024 
## Run 39 stress 0.2558769 
## Run 40 stress 0.2570361 
## Run 41 stress 0.2595734 
## Run 42 stress 0.2582375 
## Run 43 stress 0.2640813 
## Run 44 stress 0.2596681 
## Run 45 stress 0.2574303 
## Run 46 stress 0.2565465 
## Run 47 stress 0.261904 
## Run 48 stress 0.2566209 
## Run 49 stress 0.2562401 
## Run 50 stress 0.2619534 
## Run 51 stress 0.2612001 
## Run 52 stress 0.2573757 
## Run 53 stress 0.2608479 
## Run 54 stress 0.2627808 
## Run 55 stress 0.2621445 
## Run 56 stress 0.2587202 
## Run 57 stress 0.2566978 
## Run 58 stress 0.25533 
## ... Procrustes: rmse 0.01872592  max resid 0.1283567 
## Run 59 stress 0.2585886 
## Run 60 stress 0.2588408 
## Run 61 stress 0.2576459 
## Run 62 stress 0.2588744 
## Run 63 stress 0.2573896 
## Run 64 stress 0.2589411 
## Run 65 stress 0.258342 
## Run 66 stress 0.2599809 
## Run 67 stress 0.263458 
## Run 68 stress 0.2602144 
## Run 69 stress 0.2673839 
## Run 70 stress 0.2565342 
## Run 71 stress 0.2576587 
## Run 72 stress 0.2622367 
## Run 73 stress 0.2580213 
## Run 74 stress 0.2562468 
## Run 75 stress 0.2611044 
## Run 76 stress 0.2553325 
## ... Procrustes: rmse 0.01821473  max resid 0.1267855 
## Run 77 stress 0.2554911 
## ... Procrustes: rmse 0.01835597  max resid 0.1234663 
## Run 78 stress 0.2603292 
## Run 79 stress 0.2586583 
## Run 80 stress 0.2586767 
## Run 81 stress 0.2552979 
## ... Procrustes: rmse 0.01982516  max resid 0.1281269 
## Run 82 stress 0.2570593 
## Run 83 stress 0.2592984 
## Run 84 stress 0.2615321 
## Run 85 stress 0.2557913 
## Run 86 stress 0.2564797 
## Run 87 stress 0.2565491 
## Run 88 stress 0.2580999 
## Run 89 stress 0.2573066 
## Run 90 stress 0.2573345 
## Run 91 stress 0.2582829 
## Run 92 stress 0.2559852 
## Run 93 stress 0.2599086 
## Run 94 stress 0.2574749 
## Run 95 stress 0.255956 
## Run 96 stress 0.2607315 
## Run 97 stress 0.2576828 
## Run 98 stress 0.2679506 
## Run 99 stress 0.267174 
## Run 100 stress 0.260381 
## *** No convergence -- monoMDS stopping criteria:
##      2: no. of iterations >= maxit
##     98: stress ratio > sratmax
```

```
#check ordination stress and fit
ord
```

```
## 
## Call:
## metaMDS(comm = bugs, distance = "bray", k = 2, try = 100, trymax = 100,      autotransform = TRUE, expand = TRUE, plot = FALSE) 
## 
## global Multidimensional Scaling using monoMDS
## 
## Data:     wisconsin(sqrt(bugs)) 
## Distance: bray 
## 
## Dimensions: 2 
## Stress:     0.2551202 
## Stress type 1, weak ties
## No convergent solutions - best solution after 100 tries
## Scaling: centring, PC rotation, halfchange scaling 
## Species: expanded scores based on 'wisconsin(sqrt(bugs))'
```

```
summary(ord)
```

```
##            Length Class  Mode     
## nobj           1  -none- numeric  
## nfix           1  -none- numeric  
## ndim           1  -none- numeric  
## ndis           1  -none- numeric  
## ngrp           1  -none- numeric  
## diss       13203  -none- numeric  
## iidx       13203  -none- numeric  
## jidx       13203  -none- numeric  
## xinit        326  -none- numeric  
## istart         1  -none- numeric  
## isform         1  -none- numeric  
## ities          1  -none- numeric  
## iregn          1  -none- numeric  
## iscal          1  -none- numeric  
## maxits         1  -none- numeric  
## sratmx         1  -none- numeric  
## strmin         1  -none- numeric  
## sfgrmn         1  -none- numeric  
## dist       13203  -none- numeric  
## dhat       13203  -none- numeric  
## points       326  -none- numeric  
## stress         1  -none- numeric  
## grstress       1  -none- numeric  
## iters          1  -none- numeric  
## icause         1  -none- numeric  
## call           9  -none- call     
## model          1  -none- character
## distmethod     1  -none- character
## distcall       1  -none- character
## data           1  -none- character
## distance       1  -none- character
## converged      1  -none- logical  
## tries          1  -none- numeric  
## engine         1  -none- character
## species      246  -none- numeric
```

```
stressplot(ord)
```

```
goodness(ord)
```

```
##   [1] 0.02436337 0.01872328 0.02370723 0.02174370 0.02299328 0.02065903
##   [7] 0.02190949 0.01688610 0.01996983 0.01950052 0.01760799 0.01731521
##  [13] 0.01691690 0.01407678 0.01360559 0.02450168 0.02058254 0.01930925
##  [19] 0.01814806 0.02163272 0.02308586 0.02393127 0.01679506 0.01920451
##  [25] 0.01935648 0.02009092 0.01842050 0.01777458 0.01775931 0.02264793
##  [31] 0.02431124 0.02490613 0.02093369 0.01911886 0.01947060 0.01690718
##  [37] 0.02139364 0.02196927 0.02009051 0.01880069 0.01451008 0.02376348
##  [43] 0.02253602 0.01984553 0.01782923 0.02266609 0.01816223 0.02110192
##  [49] 0.01983160 0.01598197 0.01955762 0.01763593 0.01746795 0.01755212
##  [55] 0.01785664 0.01746656 0.01619652 0.01613192 0.01855065 0.01654742
##  [61] 0.01821251 0.01400531 0.01618676 0.01577785 0.02252841 0.02071953
##  [67] 0.01992242 0.02131005 0.01787321 0.01694739 0.02054749 0.01959122
##  [73] 0.02311726 0.02033289 0.01854660 0.02066839 0.02076547 0.01729727
##  [79] 0.02311251 0.02553470 0.02988479 0.02220353 0.02086767 0.02038242
##  [85] 0.01686798 0.01506480 0.01703136 0.01434554 0.02212768 0.02061784
##  [91] 0.02264952 0.01790036 0.02129025 0.01758956 0.01713818 0.01708567
##  [97] 0.02125527 0.02166508 0.02421400 0.02631600 0.01943945 0.01683794
## [103] 0.02367334 0.02217367 0.02282023 0.02262788 0.02209329 0.01849330
## [109] 0.02345670 0.02223292 0.01858378 0.02177351 0.02474453 0.02290519
## [115] 0.01956799 0.02162496 0.02121283 0.02307343 0.01929178 0.01579070
## [121] 0.02142593 0.01617426 0.02128219 0.01738216 0.02261829 0.01913726
## [127] 0.01783710 0.01822112 0.01680261 0.01961475 0.01966711 0.01893545
## [133] 0.01657174 0.01978832 0.01919173 0.01974482 0.02057027 0.02076179
## [139] 0.02076534 0.02070065 0.01939210 0.02250478 0.02392052 0.01825537
## [145] 0.01898412 0.01805221 0.02251256 0.02058423 0.01823759 0.01905687
## [151] 0.01700858 0.01792231 0.01969038 0.02243675 0.02099092 0.01893666
## [157] 0.01774734 0.02208874 0.01555742 0.01595305 0.02124233 0.01917582
## [163] 0.01459356
```

```
adonis(bugs~LZ,method="bray")
```

```
## 
## Call:
## adonis(formula = bugs ~ LZ, method = "bray") 
## 
## Permutation: free
## Number of permutations: 999
## 
## Terms added sequentially (first to last)
## 
##            Df SumsOfSqs MeanSqs F.Model      R2 Pr(>F)    
## LZ          1     4.163  4.1629  10.826 0.06301  0.001 ***
## Residuals 161    61.908  0.3845         0.93699           
## Total     162    66.071                 1.00000           
## ---
## Signif. codes:  0 '***' 0.001 '**' 0.01 '*' 0.05 '.' 0.1 ' ' 1
```

```
adonis(bugs~LZH,method="bray")
```

```
## 
## Call:
## adonis(formula = bugs ~ LZH, method = "bray") 
## 
## Permutation: free
## Number of permutations: 999
## 
## Terms added sequentially (first to last)
## 
##            Df SumsOfSqs MeanSqs F.Model      R2 Pr(>F)    
## LZH         6    14.388  2.3981  7.2383 0.21777  0.001 ***
## Residuals 156    51.683  0.3313         0.78223           
## Total     162    66.071                 1.00000           
## ---
## Signif. codes:  0 '***' 0.001 '**' 0.01 '*' 0.05 '.' 0.1 ' ' 1
```

```
adonis(bugs~S,method="bray")
```

```
## 
## Call:
## adonis(formula = bugs ~ S, method = "bray") 
## 
## Permutation: free
## Number of permutations: 999
## 
## Terms added sequentially (first to last)
## 
##            Df SumsOfSqs MeanSqs F.Model      R2 Pr(>F)    
## S          19    29.356 1.54504  6.0177 0.44431  0.001 ***
## Residuals 143    36.715 0.25675         0.55569           
## Total     162    66.071                 1.00000           
## ---
## Signif. codes:  0 '***' 0.001 '**' 0.01 '*' 0.05 '.' 0.1 ' ' 1
```

```
site.scrs <- as.data.frame(scores(ord, display = "sites"))
site.scrs <- cbind(site.scrs, Site = sega$Site)
BeetEnv <-sega[,194:203]
ef <- envfit(ord, Env, permutations = 999)
ef
```

```
## 
## ***VECTORS
## 
##                  NMDS1    NMDS2     r2 Pr(>r)    
## AvPrecip.2     0.98980  0.14247 0.3415  0.001 ***
## Precip.1      -0.95138  0.30801 0.0200  0.193    
## Temp.1        -0.98482  0.17356 0.2885  0.001 ***
## VegRich.2      0.14044  0.99009 0.0152  0.270    
## GroundCover.2 -0.13528  0.99081 0.0013  0.899    
## NDVI.2         0.97214 -0.23440 0.1727  0.001 ***
## Veg1.1         0.98100  0.19403 0.1729  0.001 ***
## Veg2.1         0.98842  0.15172 0.0552  0.012 *  
## Elevation.1    1.00000 -0.00161 0.4200  0.001 ***
## E2             0.99712 -0.07581 0.4327  0.001 ***
## Trap          -0.61466  0.78879 0.0025  0.822    
## ---
## Signif. codes:  0 '***' 0.001 '**' 0.01 '*' 0.05 '.' 0.1 ' ' 1
## Permutation: free
## Number of permutations: 999
```

```
env.scores.dune <- as.data.frame(scores(ef, display = "vectors")) #extracts relevant scores from envifit
env.scores.dune <- cbind(env.scores.dune, env.variables = rownames(env.scores.dune)) #and then gives them their names
env.scores.dune <- cbind(env.scores.dune, pval = ef$vectors$pvals)
sig.env.scrs <- subset(env.scores.dune, pval<=0.05)
nmds.plot.SEGA <- ggplot(site.scrs, aes(x=NMDS1, y=NMDS2)) +
  geom_point(aes(NMDS1, NMDS2, colour = factor(sega$LZH)))
nmds.plot.SEGA
```

```
nmds.plot.SEGA+
  geom_segment(data = sig.env.scrs, aes(x = 0, xend=NMDS1, y=0, yend=NMDS2), arrow = arrow(length = unit(0.25, "cm")), colour = "grey10", lwd=0.5) + #add vector arrows of significant env variables
  ggrepel::geom_text_repel(data = sig.env.scrs, aes(x=NMDS1, y=NMDS2, label = env.variables), cex = 4, direction = "both", segment.size = 0.25)
```
